# A Comparative Study of *N*-glycolylneuraminic Acid (Neu5Gc) and Cytotoxic T Cell (CT) Carbohydrate Expression in Normal and Dystrophin-Deficient Dog and Human Skeletal Muscle

**DOI:** 10.1371/journal.pone.0088226

**Published:** 2014-02-05

**Authors:** Paul T. Martin, Bethannie Golden, Jonathan Okerblom, Marybeth Camboni, Kumaran Chandrasekharan, Rui Xu, Ajit Varki, Kevin M. Flanigan, Joe N. Kornegay

**Affiliations:** 1 Center for Gene Therapy, The Research Institute at Nationwide Children's Hospital, Columbus, Ohio, United States of America; 2 Department of Pediatrics, The Ohio State University College of Medicine, Columbus, Ohio, United States of America; 3 Department of Physiology and Cell Biology, The Ohio State University College of Medicine, Columbus, Ohio, United States of America; 4 Departments of Medicine and Cellular and Molecular Medicine, University of California, San Diego, La Jolla, California, United States of America; 5 Department of Veterinary Integrative Biosciences, Texas A&M University, College Station, Texas, United States of America; University of Minnesota Medical School, United States of America

## Abstract

The expression of *N*-glycolylneuraminic acid (Neu5Gc) and the cytotoxic T cell (CT) carbohydrate can impact the severity of muscular dystrophy arising from the loss of dystrophin in mdx mice. Here, we describe the expression of these two glycans in skeletal muscles of dogs and humans with or without dystrophin-deficiency. Neu5Gc expression was highly reduced (>95%) in muscle from normal golden retriever crosses (GR, n = 3) and from golden retriever with muscular dystrophy (GRMD, n = 5) dogs at multiple ages (3, 6 and 13 months) when compared to mouse muscle, however, overall sialic acid expression in GR and GRMD muscles remained high at all ages. Neu5Gc was expressed on only a minority of GRMD satellite cells, CD8^+^ T lymphocytes and macrophages. Human muscle from normal (no evident disease, n = 3), Becker (BMD, n = 3) and Duchenne (DMD, n = 3) muscular dystrophy individuals had absent to very low Neu5Gc staining, but some punctate intracellular muscle staining was present in BMD and DMD muscles. The CT carbohydrate was localized to the neuromuscular junction in GR muscle, while GRMD muscles had increased expression on a subset of myofibers and macrophages. In humans, the CT carbohydrate was ectopically expressed on the sarcolemmal membrane of some BMD muscles, but not normal human or DMD muscles. These data are consistent with the notion that altered Neu5Gc and CT carbohydrate expression may modify disease severity resulting from dystrophin deficiency in dogs and humans.

## Introduction

Duchenne muscular dystrophy (DMD) is a severe X-linked myopathy of childhood that arises from the loss of dystrophin protein expression in cardiac and skeletal muscles[1], [2]. Becker muscular dystrophy (BMD) also results from mutations or deletions in the dystrophin gene, but BMD mutations allow for expression of partially functional dystrophin protein and are typically associated with phenotypically milder muscle disease than is seen in DMD. A number of animal models have been used to better define disease pathophysiology and treatments that might ameliorate DMD and BMD[3]. The model used to test most MD therapies is the mdx mouse, which lacks dystrophin in cardiac and skeletal muscles due to a nonsense point mutation in exon 23 of the dystrophin gene[4], [5]. Spontaneous mutations and deletions in the dystrophin gene also occur in dogs[6]–[8] and cats[9]–[11]. The Golden Retriever Muscular Dystrophy (GRMD) dog is one such DMD model that has been bred for research purposes[6], [12]. GRMD dogs harbor a single base pair change in the 3′ consensus splice site of intron 6, resulting in the skipping of exon 7 and an out-of-frame transcript with a stop codon immediately downstream[6], [13]. GRMD dogs model a number of features of muscle pathophysiology found in DMD, including increased muscle membrane fragility, weakened muscle force, increased force decrement in response to eccentric contractions, altered pelvic and limb joint angles arising from combined muscle hypertrophy and atrophy, increased muscle inflammation, cardiomyopathy, loss of ambulation and premature death[6], [12], [14]–[20]. In addition, treatment of GRMD dogs with corticosteroids, which can prolong ambulation in DMD[21], yields improvement in muscle outcome measures[22]. Thus, while each animal model has its own advantages, GRMD dogs are generally thought to be a very good model of the more severe aspects of DMD pathophysiology and therefore potentially more appropriate for testing DMD therapies.

It is clear from a number of recent studies that glycosylation of the sarcolemmal membrane with particular carbohydrate structures can impact muscle membrane integrity and disease[23]–[27]. One such modifier of disease severity in the mdx mouse model is the *Cmah* gene[28]. *Cmah* encodes the CMP-Neu5Ac hydroxylase, the only enzyme known to hydroxylate the sugar nucleotide precursor for sialic acids at the 5-N-acyl position, converting CMP-Neu5Ac to CMP-Neu5Gc[29], [30]. The twenty or so sialyltransferases in mammals utilize these substrates to add sialic acids to glycoproteins and glycolipids[31]. In all mammals studied to date, save humans, *Cmah* encodes an active enzyme that can result in Neu5Gc on glycoproteins and glycolipids. In mouse skeletal and cardiac muscle, Neu5Gc and Neu5Ac are the predominant sialic acid forms[28]. About 2–3 million years ago, after the divergence of humans from the great apes, a 92 base pair deletion in exon 6 of the human *CMAH* gene led to its inactivation from a resulting frame shift[32], and as a result all humans lack the biosynthetic ability to create Neu5Gc[33], [34]. Deletion of mouse *Cmah* to reiterate this event in human evolution does not grossly impact muscle function, but loss of *Cmah* in mdx mice leads to more severe disease pathology, resulting in additional reductions in the strength of diaphragm and cardiac muscles as well as reduced lifespan[28]. While many other modifiers of disease severity occur in mdx mice, for example integrin α7[35], utrophin[36], [37], MyoD[38], sarcospan[39], [40] and telomerase[41], this is the first example of a disease modifier where the genetic change actually reiterates a human-specific genetic event.

As *Cmah^−/−^*mdx mice show more severe disease severity than mdx animals, the question arises as to how Neu5Gc may impact disease in other animal models, such as dogs and cats. One comparative study of muscle glycolipids has shown that dog muscle has very low Neu5Gc levels, with Neu5Gc comprising 2% of total sialic acid as compared to much higher levels in the mouse, cow and monkey[42]. This raises the possibility that dog muscles are hypomorphic for Neu5Gc, even though they appear to express a functional canine *CMAH* gene.

Another carbohydrate structure that can modify muscular dystrophy is the Cytotoxic T cell (CT) carbohydrate (GalNAcβ1-4[Neu5Ac/Gcα2-3]Galβ1-4GlcNAc-)[43]–[45]. The CT carbohydrate is normally confined to the neuromuscular junction in adult mouse and human skeletal muscle, with additional expression in intramuscular capillaries[46], [47]. The enzyme that defines the synaptic expression of the CT carbohydate is encoded by the *Galgt2* gene (also called *b4Galnt2*). *Galgt2* encodes a β1-4GalNAc glycosyltransferase that creates the terminal β1-4GalNAc linkage on the CT carbohydrate[48]–[50]. Overexpression of *Galgt2* in mouse skeletal myofibers stimulates the glycosylation of α dystroglycan with the CT carbohydrate and induces the ectopic overexpression of normally synaptic dystroglycan-binding proteins, including utrophin, laminin α4, laminin α5 and agrin[48], [51]–[56]. Such overexpression of Galgt2 can ameliorate muscular dystrophy in three different mouse models of muscular dystrophy, the mdx model for DMD[55], the *Sgca^−/−^* model for LGMD2D[53] and the dy^W^ model for MDC1A[52]. *Galgt2* overexpression correlates with increased laminin and agrin binding to α dystroglycan[54] and with increased resistance of skeletal muscles to injury resulting from eccentric contractions[24].

Despite the demonstrated functional effects of these carbohydrates on muscle disease, there is no published information on CT carbohydrate expression and little to no information on Neu5Gc expression in BMD or DMD muscles or in golden retriever (GR) or GRMD muscles. As low levels of Neu5Gc and CT carbohydrate correlate with more severe disease in mice, we asked here whether a similar relative deficit might potentially contribute to worsened disease severity in dystrophin-deficient dog and human muscles.

## Materials and Methods

### Ethics Statement

mdx and wild type (both in a C57Bl/10 genetic background) mice were bred at Nationwide Children's Hospital in accordance with protocols approved by the Institutional Animal Care and Use Committee and specifically approved for use in this study (Permit AR07-00033). The full name of this committee is the “Institutional Animal Care and Use Committee at Nationwide Children's Hospital”.

GR crosses and GRMD dogs were bred at University of North Carolina, Chapel Hill, in accordance with protocols approved by their Institutional Animal Care and Use Committee under a protocol that included the experiments described in this study (Permit 06-338). The full name of this committee is the “Institutional Animal Care and Use Committee at the University of North Carolina”.

Blinded sections from human muscle biopsies were obtained from clinical specimens archived as part of the United Dystrophinopathy project in accordance with approval from the Institutional Review Board (IRB) at Nationwide Children's Hospital. The full name of this committee is the “Institutional Review Board at Nationwide Children's Hospital”. All samples were obtained with patient's written informed consent. As part of the consent process approved by the IRB, we received written consent from guardians on behalf of children/minors. Dated and signed consent forms were archived.

### Mice

mdx and wild type (both in a C57Bl/10 genetic background) mice were bred at Nationwide Children's Hospital in accordance with protocols approved by the Institutional Animal Care and Use Committee. mdx mice were originally obtained from Jackson Laboratory and *Cmah^−/−^* mice and *Cmah^−/−^*mdx mice were generated previously[28], [57]. Muscles from mdx mice treated with Adeno associated virus (AAV) vector to overexpress the human *GALGT2* gene (also termed *B4GALNT2*), using rAAV(rh.74).MCK.GALGT2, were utilized from a previous study[24].

### Dogs

GR crosses and GRMD dogs were bred at University of North Carolina, Chapel Hill, in accordance with protocols approved by their Institutional Animal Care and Use Committee. Assessment of severity of muscle disease was measured by observation of gait and ambulation as well as measures of tibiotarsal joint (TTJ) angle and maximal tibiotarsal flexion and extension, per previous studies[14], [16], [19]. For sake of defining disease severity in these GRMD dogs, we characterized several phenotypic features. Ringo and Napoleon were characterized as “severe” based on TTJ extension tetanic force and TTG angles below 1 N/kg and 145°, respectively, while values above these levels were associated with a “mild” phenotype in Tico, Jane and Summer (Table S1). Biopsy or necropsy samples of cranial sartorius (CS) and vastus lateralis (VL) muscles were serially obtained at 3, 6 and 13 months of age. Methods to measure muscle physiology and other measures describing dystrophic changes have been described in previous studies[16], [19].

### Human muscle biopsies

Blinded sections from human muscle biopsies were obtained from clinical specimens archived as part of the United Dystrophinopathy project in accordance with approval from the Institutional Review Board. Normal human muscles were from a 55 year-old (yo) male, a 5yo male and a 6yo female; DMD muscles were taken from 6 and 5yo males and BMD muscles from 11yo, 35yo and 67yo males. Biopsies were taken from the quadriceps muscle in most instances. “Normal” human samples were defined as biopsies where no myopathy or evidence of inflammation was present. For all normal cases, dystrophin protein expression was verified as being positive, and for all DMD cases, dystrophin protein expression was verified as being negative. DMD cases arose from a duplication of exons 29–43, c.3603+2T>C splice site mutation, or a duplication of exons 3–6. The three BMD cases arose from an intron 11 c.1331+17770C>G pseudoexon mutation (leading to mutant RNA r.1331_1332ins1331+17691_1331+17769 (case BZ), with loss of ambulation at age 23, an exon 2 c.40_41delGA frameshift mutation, with ambulation until at least 36 (case KG), and an intron 25 c.3432+3730G>T pseudoexon mutation, leading to mutant RNA r.3432_3433ins3432+3663_3432+3728, with ambulation until at least 67 (case RS). None of the cases were treated with steroids prior to biopsy.

### Immunostaining

Human muscle biopsies and mouse skeletal muscles were snap frozen in liquid nitrogen-cooled isopentane; dog muscle biopsies were frozen in liquid nitrogen-cooled SUVA34A (Freon analogue). 8 µm cross-sections of all skeletal muscles were cut on a cryostat. For staining with antibody to glycans bearing *N*-glycolylneuraminic acid (Neu5Gc, affinity purified chick IgY; Sialix) or non-immune chick IgY control (Sialix), sections were blocked in phosphobuffered saline (PBS) with 10% human serum that was confirmed to be Neu5Gc-free. Sections were incubated with anti-Neu5Gc or control antibody (1∶500 or 1∶1000) in blocking solution (10% human serum) overnight at 4°C, washed in PBS, incubated with donkey anti-chicken IgY secondary antibody conjugated to FITC (1∶250; 703-485-155, Jackson Immunoresearch), washed again in PBS and mounted in glycerol with paraphenylenediamene to inhibit fluorescence quenching. For *Maackia amurensis* agglutinin (MAA) staining, sections were blocked in 5% bovine serum albumin (BSA) in PBS. Sections were incubated with FITC-conjugated MAA (10 µg/mL, EY laboratories, F-7801), washed in PBS and then mounted. All MAA staining could be blocked by addition of exogenous excess sialic acid to demonstrate lectin-binding specificity. Anti-Neu5Gc immunostaining could be blocked with either 10% chimpanzee serum, which has excess Neu5Gc, or by pre-treating sections with mild periodate to destroy the immunogenic side chain of Neu5Gc. None of these methods, or staining with non-immune chick IgY serum, differed from the control images shown. *Wisteria floribunda* agglutinin (WFA) linked to FITC was used to stain muscle at 2 µg/mL (EY Laboratories, F-3101), with 5% BSA in PBS used as a blocking agent. CT1 and CT2 staining was performed as previously described using antibody purified from hybridoma supernatant[58]. Staining for CT1, CT2 or control anti-mouse IgM(only) was blocked in 3%(v/v) BSA in PBS for one hour, followed by incubation with hybridoma supernatant (used straight or diluted 1∶2) overnight. Sections were then washed in PBS and incubated in goat anti-mouse IgM(only) conjugated to Cy2 (115-225-020, Jackson ImmunoResearch).

For double immunostaining, sections were first stained overnight at 4°C with anti-Neu5Gc or control-specific chicken IgY after blocking in 10% (Neu5Gc-free) human serum, or with CT1 or CT2 after blocking in 5 mg/mL BSA, as described above. For Pax7 co-staining, sections were fixed in 2% paraformaldehyde. After blocking in 10% human serum, sections were then incubated overnight mouse-anti Pax7 (clone P3U1, Developmental Studies Hybridoma Bank), followed by species- and isotype-specific fluorophore-conjugated secondary antibodies for one hour. Pax7 antibody was a generous gift from Michael Rudnicki (Ottawa Health Research Institute). For all other anti-Neu5Gc or anti-CT co-stains, sections were incubated overnight with both primary antibodies without fixation, washed for one hour and incubated with the appropriate secondary antibodies as above. All sections were washed and placed in mounting medium containing with DAPI. Co-staining antibodies used in dog were mouse anti-chicken Pax7 (Developmental Studies Hybridoma Bank, clone P3U1), mouse anti-dog CD4 (AbD Serotec, MCA1998S), rat anti-dog CD8 (AbD Serotec, MCA1039GA), mouse anti-dog CD11b (AbD Serotec, MCA1777S), mouse anti-dog CD21 (AbD Serotec, MCA1781R), mouse anti-human β spectrin (Novus, NB300-574) or mouse anti-rat embryonic myosin (NovaCastra, NCL-MHCd). Co-staining antibodies used in human were mouse anti-chicken Pax7 (Developmental Studies Hybridoma Bank, clone P3U1), FITC-conjugated rabbit anti-human CD4 (BD Bioscience, 550628), FITC-conjugated mouse anti-human CD8 (AbD Serotec, MCA1039GA), rabbit anti-human CD11b (Abcam, ab52478), mouse anti-human β spectrin (Abcam, ab2808), mouse anti-rat embryonic myosin (eMyosin; NCL-MHCd, NovaCastra), rabbit anti-human calnexin (Sigma, C4731), rabbit anti-human clathrin (Cell Signaling, P1663), rabbit anti-human LAMP1 (Sigma, L1418) or mouse anti-human 58K Golgi protein (Novus, NB600-412). Other sections were co-stained with WFA-FITC and mouse anti-rat embryonic myosin (eMyosin; NCL-MHCd, NovaCastra). Appropriate fluorophore-conjugated species or species- and isotype-specific secondary antibodies were obtained from Jackson Immunoresearch. Imaging was done on a Zeiss Axiophot epifluorescence microscope using AxioVision LE 4.1 imaging software (Zeiss; Jena, Germany) with fluorescein-, DAPI-, or rhodamine-specific optics. AAV(rh.74).MCK.*GALGT2*(human)-treated mdx muscles were infected for 12 weeks, as previously described[24]. All images shown in a figure use time-matched exposures for comparison and were representative of commonly seen staining patterns. For quantification of cell staining, 5 random 10x images per muscle were taken and counted as previously described[59].

### Western blotting

Western blots for Neu5Gc and glyceraldehyde 3-phosphate dehydrogenase (GAPDH) were done and quantified as previously described using 15 or 30 µg per lane of NP40-extracted skeletal muscle protein separated on by SDS-PAGE on a 4–12% gradient gel[60].

### Quantification of sialic acids

Quantification of total sialic acid and sialic acid species (relative Neu5Gc and Neu5Ac levels) was performed with modification from previously described methods of DMB (1,2-diamino-4,5-methylenedioxybenzene dihydrochloride) derivatization[61]. Briefly, 20–60 mg muscle tissue was homogenized in 10% PBS, base treated with 100 mM NaOH at 37°C for 30 minutes, then neutralized with 100 mM HCl. Small aliquots were taken for protein concentration determination using a Bradford assay. Sialic acids were then hydrolyzed with 2 M acetic acid at 80°C for 3 hours and passed through a 10 K molecular weight filter. Flow-through was concentrated and subjected to DMB derivatization at 50°C for 2.5 hours. Samples were then resolved using high performance liquid chromatography (HPLC) and Neu5Ac and Neu5Gc peaks quantified, as before[47].

## Results

We chose to study two dog muscles, the vastus lateralis (VL) and the cranial sartorius (CS), because these muscles have been well documented to undergo differential changes relevant to muscular dystrophy in GRMD animals[17]. In particular, the CS shows more muscle necrosis during the neonatal period and subsequent hypertrophy by 6 months of age, while the VL has a more delayed pattern of muscle necrosis and regeneration that leads to atrophy (Figure S1). Muscle biopsies or necropsies from three GR and five GRMD dogs were used for this study. Of the five GRMD dogs, two (Ringo and Napoleon) showed severe muscular dystrophy by six months of age, as evidenced by decreased tibiotarsal joint angle and decreased force of tibiotarsal joint extension, two well-described measures of GRMD disease severity[16]–[18], [62], while three others (Tico, Jane and Summer) were mildly affected, having measures for these parameters that neared or equaled wild type dogs despite having the same GRMD genetic defect (Table S1).

**Figure 1:**
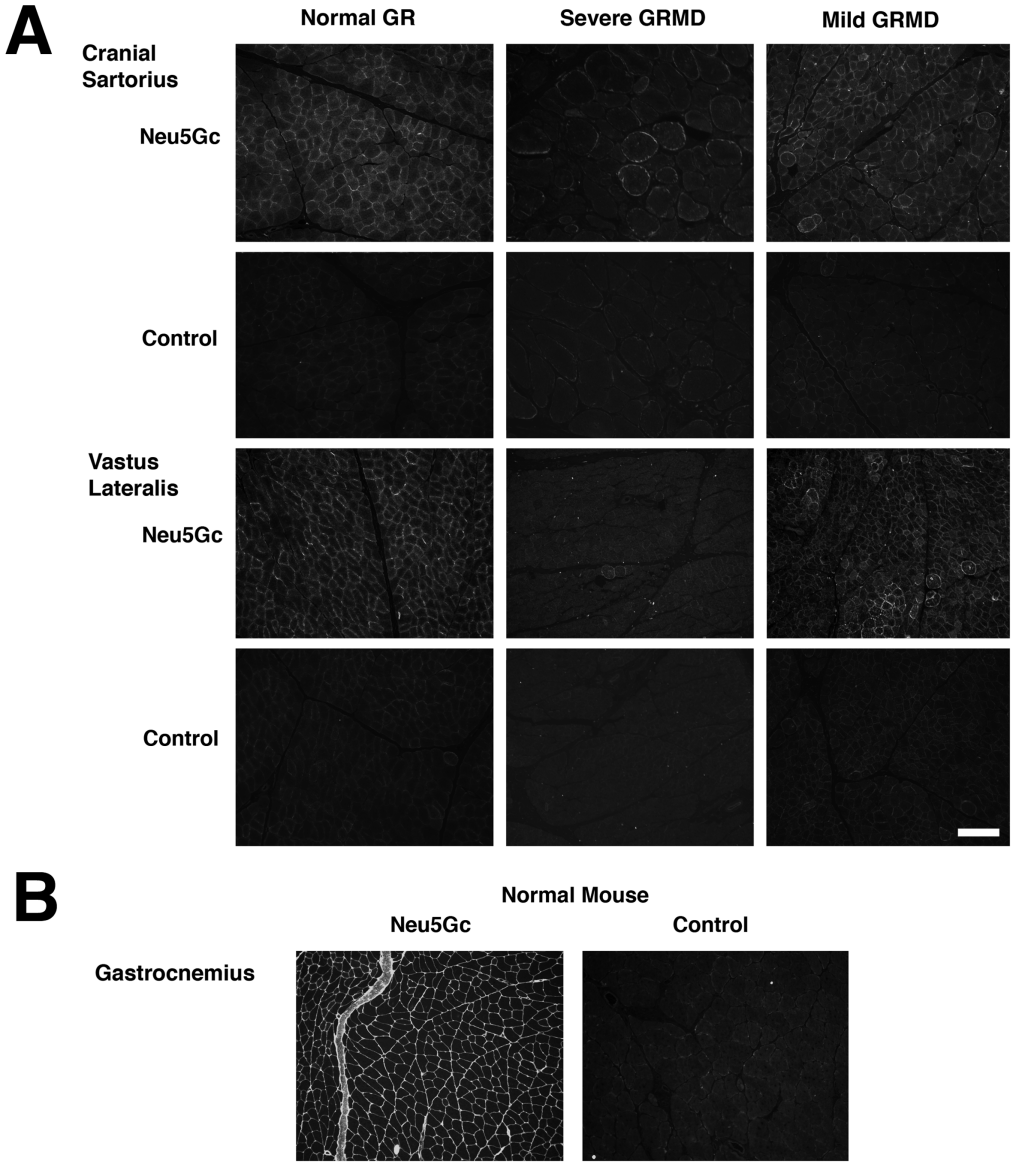
Neu5Gc expression in Golden Retriever cross (GR) and Golden Retriever Muscular Dystrophy (GRMD) dog skeletal muscle relative to mouse. (A) Neu5Gc-specific affinity-purified chicken IgY was used to stain skeletal muscles from 6 month-old normal Golden Retriever (GR) or Golden Retriever Muscular Dystrophy (GRMD) dogs. An example of staining from a severely affected and a mildly affected GRMD dog are shown. (B) Time-matched images in A were compared to normal mouse skeletal muscle immunostained with the same reagents. Non-immune chicken IgY was used as a control for background staining in A and B. Bar is 200 µm for all panels in A and B.

We first compared Neu5Gc staining in muscles from 6 month-old GR (Swiper), mildly affected GRMD (Tico) and severely affected GRMD (Ringo) dogs (Figure 1A). As expected from the previously documented low level of Neu5Gc in dog muscle gangliosides[42], we found very little Neu5Gc staining in GR or GRMD muscle membranes. In addition, there was no Neu5Gc immunostaining of intramuscular connective tissue in any dog muscle (Figure 1A). There was some difference in fiber type between different GR and GRMD muscles, with CS showing a bias towards more slow fibers than the VL, as has been previously described[62]. Positively stained GR myofibers showed an uneven pattern of membrane staining, with some myofibers showing no staining, while GRMD muscles appeared to have even lower levels of staining than GR muscles (Figure 1A). While Neu5Gc staining of muscles from the severely affected GRMD dog appeared to be reduced relative to staining of the muscles from the mildly affected GRMD dog, this was not found to be significant when comparing all sections stained from all GRMD animals (not shown). By contrast, anti-Neu5Gc immunostaining of normal mouse gastrocnemius muscle showed very high and uniform staining of skeletal myofibers as well as intramuscular connective tissue (Figure 1B). Staining of muscles with pre-immune chicken IgY (Figure 1A and 1B, control) or secondary antibody alone (not shown) showed no background immunostaining. Thus, both GR and GRMD muscles showed very little Neu5Gc expression.

**Figure 2:**
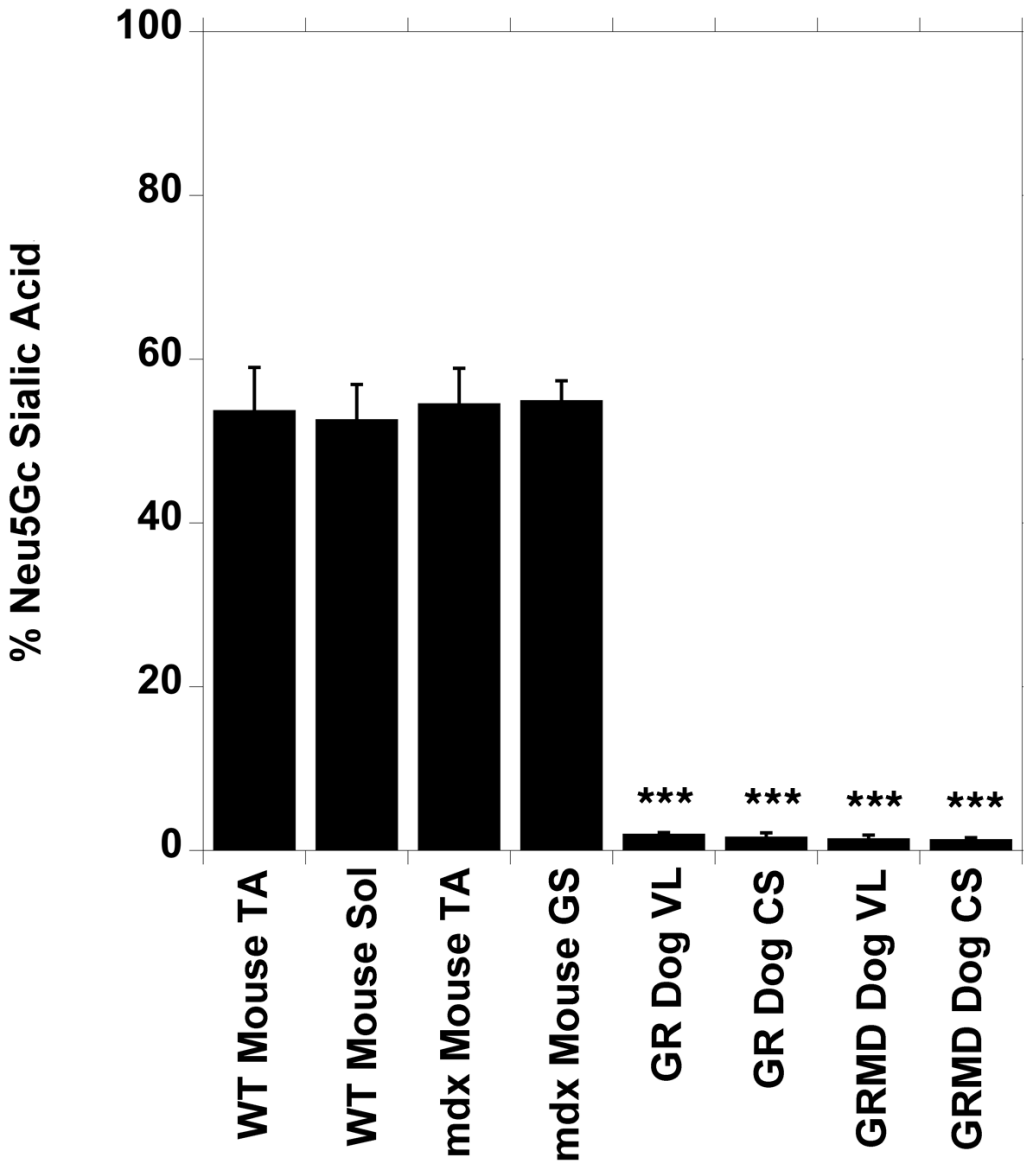
Quantification of Neu5Ac and Neu5Gc levels in normal and dystrophin-deficient mouse and dog muscles. Tibialis anterior (TA) and soleus (Sol) muscles were analyzed from 11 wild type (WT) mice, TA and gastrocnemius (GS) muscles were analyzed from 8 mdx mice, and vastus lateralis (VL) and cranial sartorius (CS) muscles were analyzed from 2 golden retriever (GR) and 5 golden retriever muscular dystrophy (GRMD) dogs. Average Neu5Gc as a percentage of total sialic acid is shown. Errors are standard deviation (SD). ***P<0.001, for all dog vs. mouse muscle comparisons.

To investigate this further, we determined Neu5Gc levels, as a fraction of total sialic acids, in skeletal muscles from wild type and mdx mice and from GR and GRMD dogs (Figure 2). In addition, we compared Neu5Gc expression on GR and GRMD muscle glycoproteins to muscles from wild type (*Cmah^+/+^* and *Cmah^−/−^*) mice (Figure S2). In both instances, both GR and GRMD muscles (CS and VL) expressed a fraction of the signal seen in mouse muscles (tibialis anterior, soleus and gastrocnemius). Neu5Gc levels did not exceed 2% of total sialic acids for any GR and GRMD muscle, while Neu5Gc levels in wild type and mdx mouse muscles averaged 52–55% (Figure 2). Thus, Neu5Gc levels in all GR and GRMD muscles were significantly reduced, by at least 95%, relative to mouse muscles (P<0.001, any dog muscle compared to any mouse muscle in Figure 2). Additionally, pooled GRMD muscles had a significant reduction in Neu5Gc relative to GR (1.9±0.2% of total sialic acid in GR vs. 1.4±0.1% in GRMD, P<0.05). Total sialic acid content per mole protein, however, was not reduced in GRMD muscles compared to GR (not shown). Reduced Neu5Gc on glycoproteins was evident for both severely affected and mildly affected GRMD muscles (Figure S2). For example, Neu5Gc blot signals from all GRMD VL samples was 3±2% of mouse *Cmah^+/+^* muscle signal and 16±6% of GR VL signal. The low number of GRMD dogs precluded us from making definitive associations between muscle Neu5Gc levels and individual (severe versus mild) GRMD phenotypes.

To assess overall sialic acid expression, we also stained muscle sections from GR and GRMD animals with *Maackia amurensis* agglutinin (MAA), a sialic acid-binding lectin that recognizes sialic acid in a α2-3-linkage[63](Figure S3). GR and GRMD muscles, from both CS and VL, showed high levels of MAA staining along skeletal myofiber membranes and within intramuscular connective tissue. Increased fibrosis was evident from MAA staining of GRMD CS in a severely affected dog, as was the presence of hypertrophic skeletal myofibers. Thus, the low expression of Neu5Gc in GR and GRMD muscle was not due to a reduction in overall sialic acid levels.

**Figure 3:**
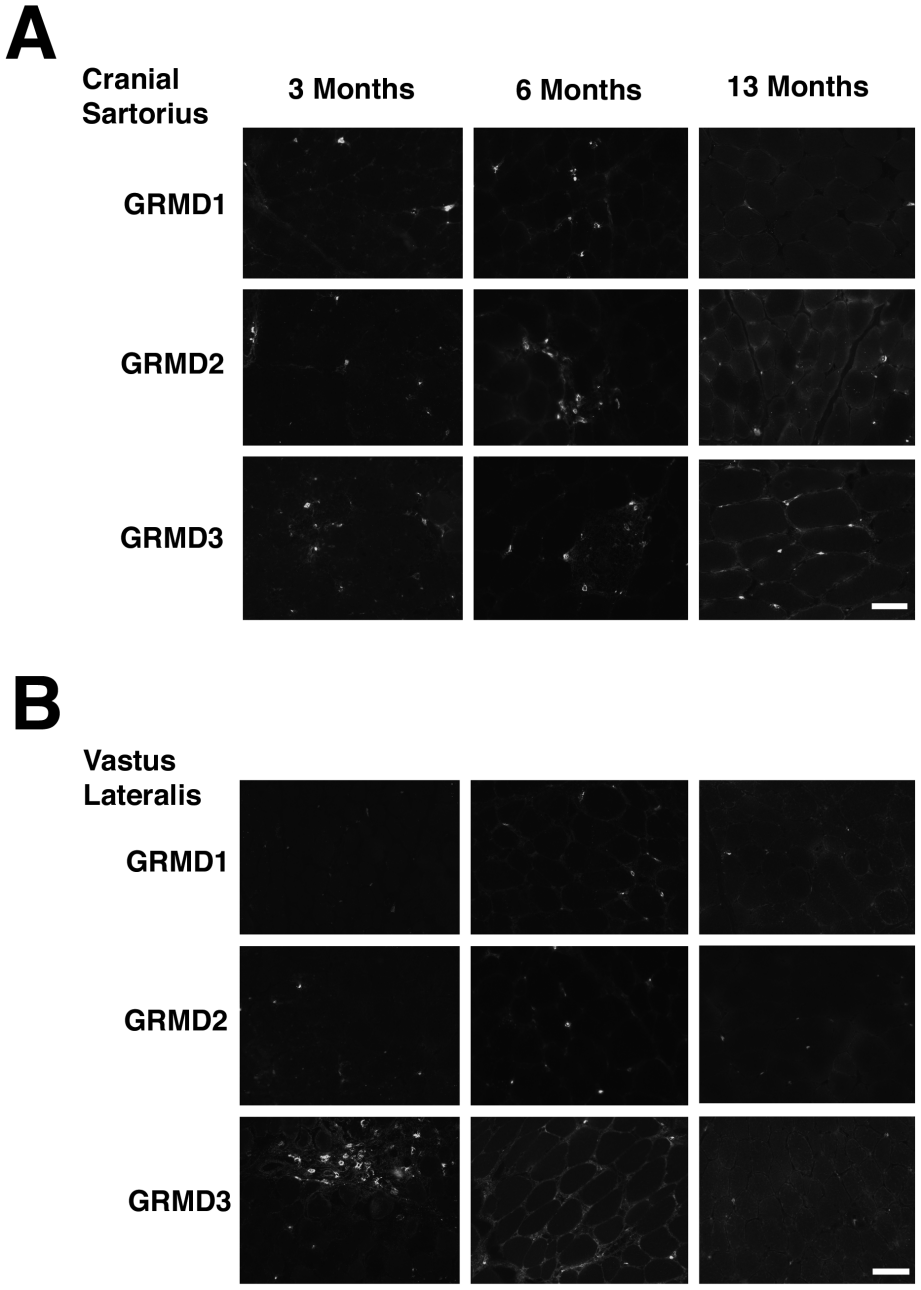
Neu5Gc expression in GRMD muscle at different ages. Three different GRMD cases (GRMD1, 2, and 3) were biopsied and muscles stained for Neu5Gc at 3, 6 and 13 months of age. Time-matched images of staining of the cranial sartorius (A) and vastus lateralis (B) muscles are shown. Bar is 50 µm for all panels in A and B.

We next performed a longitudinal study of anti-Neu5Gc staining for three different GRMD dogs, imaged at higher magnification, to assess expression at 3, 6 and 13 months of age (Figure 3). Muscles in all three GRMD dogs (GRMD1-3, Napoleon, Jane and Summer, respectively) showed higher Neu5Gc staining in mononuclear cells within the muscle than was seen on GRMD myofibers. There was faint Neu5Gc expression in some GRMD skeletal myofibers at 6 and 13 months of age compared to 3 months, however, this staining was not uniformly present. We stained the same GRMD dog muscles with MAA to assess relative total α2-3-linked sialic acid levels (Figure S4). MAA staining was high and relatively constant in both CS and VL muscles of GRMD dogs at 3, 6 and 13 months of age. MAA staining showed evidence of increased hypertrophic myofibers in the CS versus VL at 6 and 13 months, consistent with previous studies[17].

**Figure 4:**
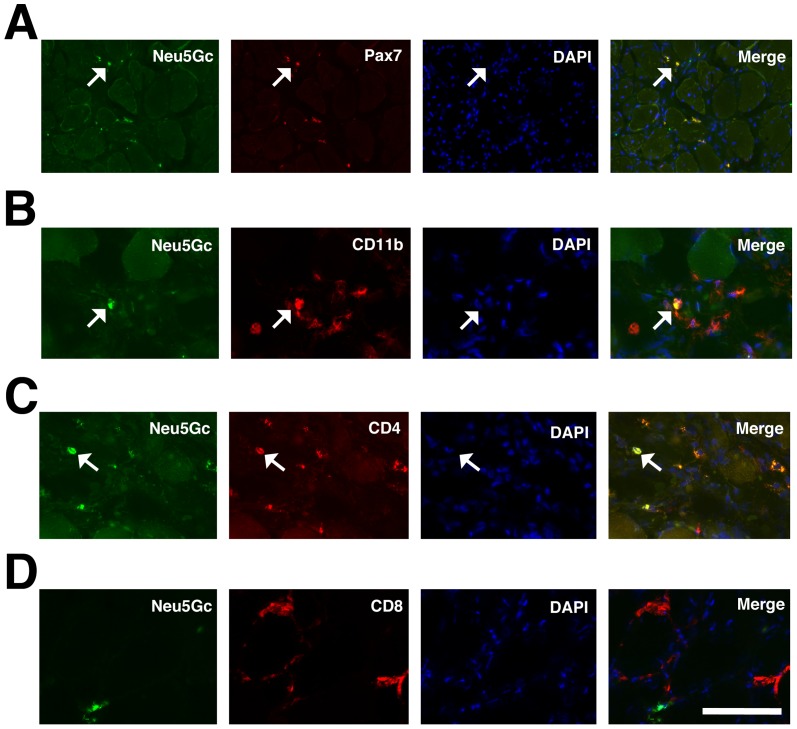
Neu5Gc immunostaining of satellite cells, T lymphocytes and macrophages in GRMD skeletal muscle. GRMD muscle (cranial sartorius) was triple stained for Neu5Gc (green), Pax7 (A), CD11b (B), CD4 (C), CD8 (D) (all red), and DAPI (blue). Arrows indicated co-staining of Neu5Gc and Pax7 (in A), CD11b (in B) or CD4 (in C). Bar is 100 µm (A) and 50 µm (B–D) for all panels.

**Figure 5:**
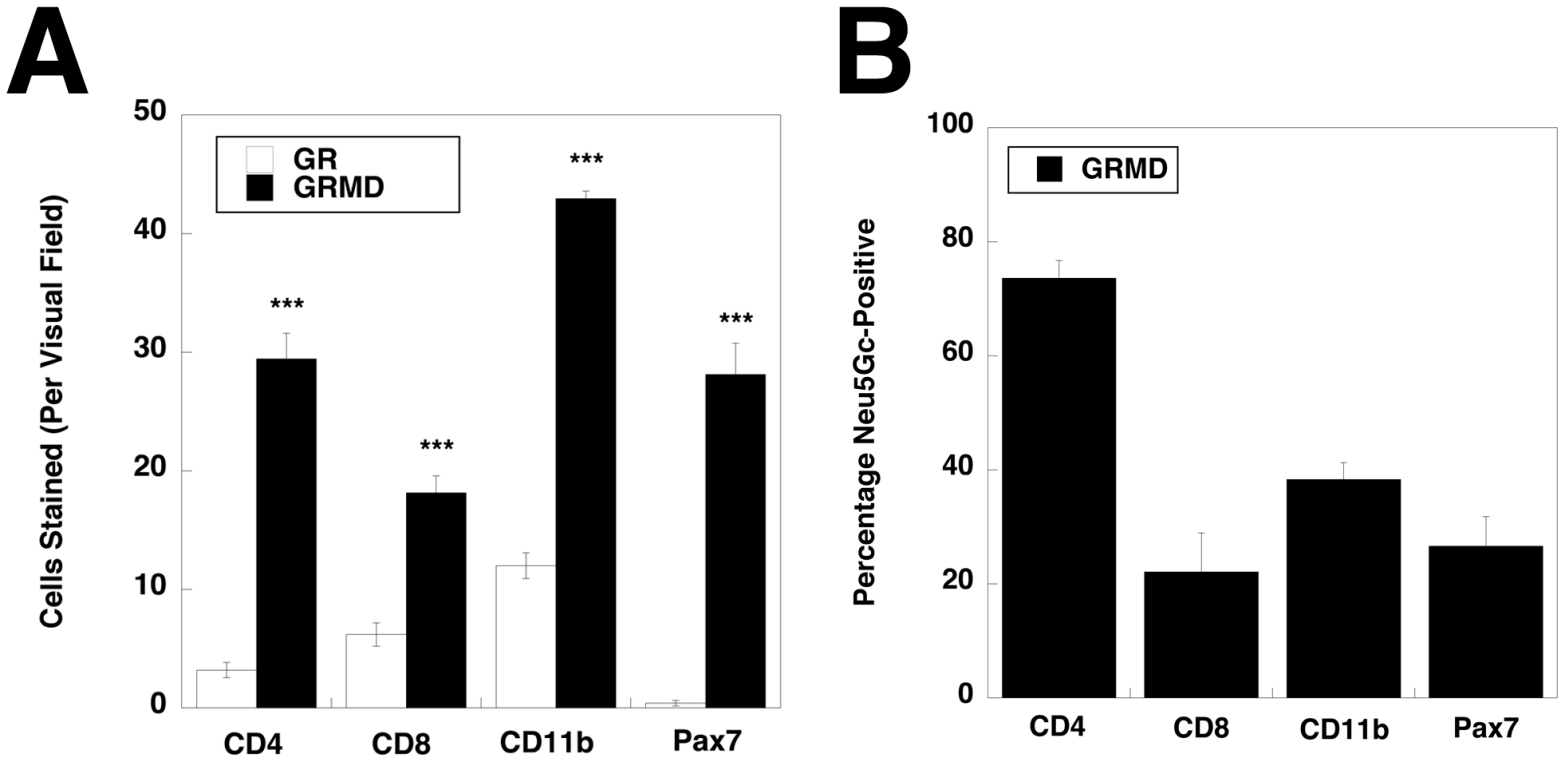
Quantification of CD4−, CD8−, CD11b− and Pax7−stained cells in GR and GRDM muscle and of Neu5Gc co-staining in GRMD muscle. (A) Cranial sartorius muscle sections from GR and GRMD dogs were stained with markers for satellite cells (Pax7), T lymphocytes (CD4 or CD8) or macrophages (CD11b) and quantified for numbers of cells stained per 40X visual field. (B) The percentage of cells co-stained for Neu5Gc and CD4, CD8, CD11b or Pax7 in GRMD muscles was quantified. Errors are SEM. ***P<0.001, for each GR vs. GRMD comparison in A.

To further delineate which mononuclear cells expressed Neu5Gc in GRMD muscles, we triple stained muscle sections for Neu5Gc, DAPI (to stain nuclei) and markers for Pax7 (Figure 4A), to identify satellite cells, CD11b (Figure 4B), to identify macrophages, CD4 (Figure 4C), to identify helper T lymphocytes, or CD8 (Figure 4D), to identify cytotoxic T lymphocytes. We utilized mouse hybridoma clone P3U1 for Pax7 staining. A subset of cells that stained for Neu5Gc could be co-stained with each cell-specific marker used (except CD21, not shown, which did not stain muscle). Because each of these cell types co-expressed Neu5Gc, we quantified both the total number of each cell type per unit area (Figure 5A) as well as the percentage of each cell type that co-expressed Neu5Gc (Figure 5B). GRMD muscles showed higher numbers of Pax7−, CD4−, CD8− and CD11b−stained cells compared to GR muscles. Aside from CD4+ cells, however, all cell types showed a low percentage of co-expression with Neu5Gc in GRMD muscles, with only a quarter to half of Pax7+, CD8+ or CD11b+ cells co-expressing Neu5Gc (Figure 5B). For CD8 and Pax7, the percentage of cells expressing Neu5Gc in GRMD muscle was also significantly lower than it was in GR muscle (not shown).

**Figure 6:**
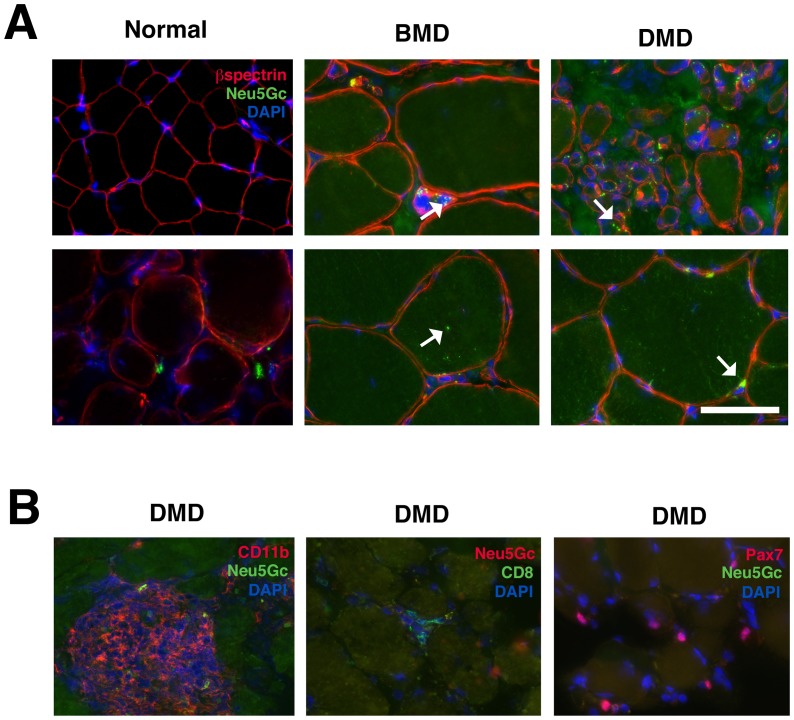
Neu5Gc co-staining with β spectrin, CD11b, CD8 or Pax7 in normal, BMD or DMD human muscle. (A) Otherwise normal, Becker muscular dystrophy (BMD) and Duchenne muscular dystrophy (DMD) muscle biopsy sections were stained for Neu5Gc (green), β spectrin (red) and DAPI (blue). Arrows indicate Neu5Gc puncta in cytoplasmic or perimembranous regions of BMD and DMD skeletal myofibers. (B) DMD muscle was co-stained for Neu5Gc and CD11b, CD8 or Pax7 (and DAPI). Bar is 50 µm for all panels in A and B.

**Figure 7:**
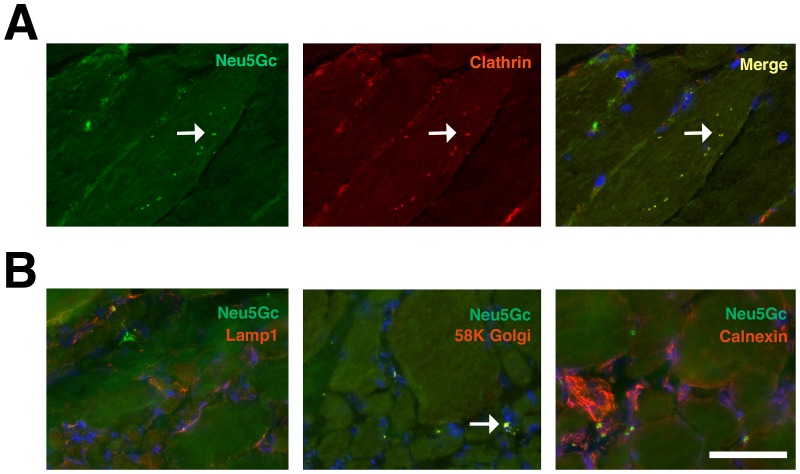
Co-localization of Neu5Gc staining with markers for endosomes and Golgi in BMD and DMD muscle. (A) BMD muscle co-stained for Neu5Gc (green) and clathrin (red), a marker of endosomes. Merged image on right shows overlap of Neu5Gc and clathrin expression in yellow. Arrow marks several examples of co-staining. (B) DMD muscle co-stained for Neu5Gc (green) with LAMP1, a lysosomal marker, 58K Golgi, a Golgi marker, or calnexin, and endoplasmic reticulum marker, all in red, and DAPI (blue). Arrow marks region of coincident staining (yellow) for Neu5Gc and 58K Golgi. Bar is 50 µm for all panels in A and B.

We next analyzed Neu5Gc staining of muscle sections from biopsies of patients with Becker muscular dystrophy (BMD) or Duchenne muscular dystrophy (DMD) (Figure 6 Figure S5). These were compared to muscle sections from patients with an initial complaint that warranted muscle biopsy but where no muscle pathology could be identified (otherwise “normal” human muscle). We identified no Neu5Gc staining in normal human muscle, consistent with previous studies[28]. Unlike previously published Neu5Gc staining[28], however, we also identified some punctate intracellular Neu5Gc staining in BMD and DMD muscles. This was the case in all three DMD and all three BMD biopsies analyzed (Figure S5). Co-staining of Neu5Gc and β spectrin, a sarcolemmal membrane marker, showed that most Neu5Gc staining was localized within skeletal myofibers or near the sarcolemmal membrane, with greater concentrations being found within smaller myofibers (Figure 6A). By contrast, there was minimal to no co-staining of Neu5Gc with CD11b, CD8 or Pax7 in DMD muscle (Figure 6B). The intracellular aggregates of Neu5Gc within skeletal myofibers often co-stained with clathrin, a marker for endosomes (Figure 7A). In addition, some co-staining was evident with 58K Golgi protein, a marker for the Golgi apparatus (Figure 7B). There was minimal co-staining with LAMP1, a marker for lysosomes, or with calnexin, a marker for the endoplasmic reticulum (Figure 7B). Thus, the majority of Neu5Gc-stained puncta found within skeletal myofibers were co-stained with a marker for endosomes (Figure 7), suggesting that Neu5Gc is internalized by endocytosis into dystrophin-deficient human muscles and concentrated in distinct intramuscular and perimembranous regions of skeletal myofibers.

**Figure 8:**
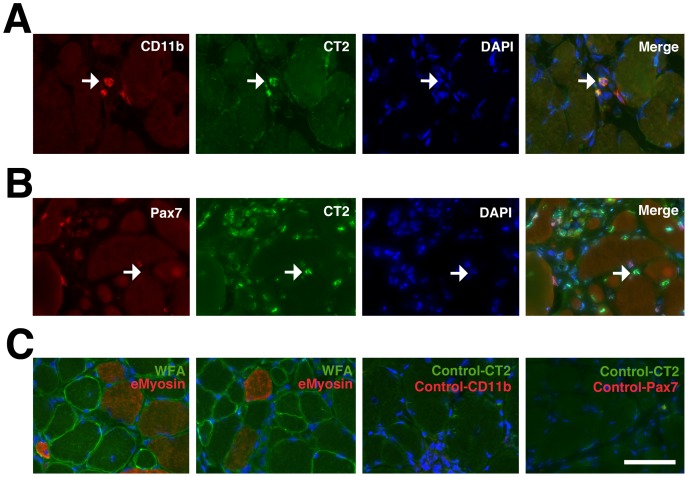
Co-staining of CT carbohydrate with macrophages in GRMD muscle. (A) Single and merged staining for CT2 (green) with CD11b (red, to mark macrophages) and DAPI (blue). Arrow shows co-incident staining for CT2 and CD11b. (B) Single and merged staining for CT2 (green) with Pax7 (red, to mark satellite cells) and DAPI (blue). Arrow shows lack of coincident staining for CT2 and Pax7. (C) Examples of WFA staining (green) with embryonic myosin (red) and DAPI (blue) (two left panels). Examples of control staining for staining shown in A and B using only secondary antibodies with DAPI (two right panels). Bar is 50 µm for all panels in A, B and C.

We next surveyed CT carbohydrate and overall βGalNAc expression in GR and GRMD muscles, using the CT2 monoclonal antibody and *Wisteria floribunda* agglutinin (WFA), respectively (Figure 8 and Figure S6). In GR muscle, most WFA and CT2 staining was present at the neuromuscular junction (NMJ) (Figure S5). NMJs were identified by costaining with rhodamine-α-bungarotoxin, which labels nicotinic acetylcholine receptors (AChRs). In GRMD muscle, we also identified increased WFA staining on extrasynaptic regions of the muscle sarcolemmal membrane, as described previously in mdx mice[64]. WFA staining was not as abundant in eMyosin-positive myofibers as in eMyosin-negative myofibers (Figure 8C). In addition, we identified intense CT2 staining in mononuclear cell infiltrates in GRMD muscle. Here, CT2 co-stained with CD11b, suggesting that much of this CT expression was present on macrophages (Figure 8A). While CT2 co-stained large aggregates of macrophages in some muscle regions, an example with only a few macrophages present is shown here for clarity. CT2 co-stained with Pax7 far less frequently than with CD11b (Figure 8B). These data are similar to immunostaining results in mdx muscle[55] and show that CT carbohydrate expression can be increased in GRMD muscle relative to GR.

**Figure 9:**
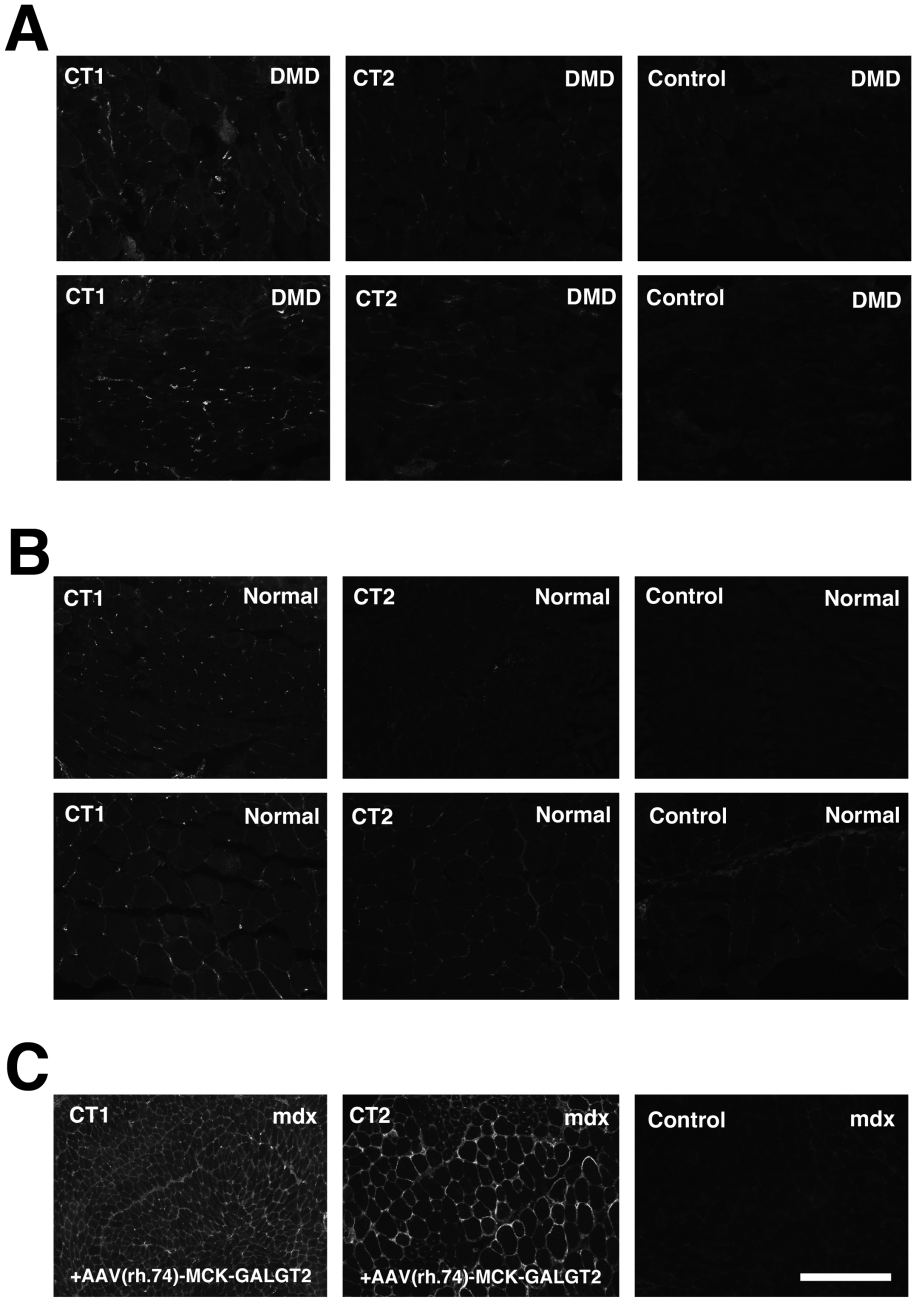
Comparison of CT1 and CT2 immunostaining in DMD and normal human muscle. CT1 and CT2 were used to immunostain two different DMD (A) and two different normal human (B) muscle biopsies, compared to secondary antibody control. These were compared to time-matched images taken from mdx muscles (C), diaphragm (left panel) or tibialis anterior (right panel), that had been infected with rAAV(rh.74).MCK.GALGT2 for 12 weeks. Bar is 200 µm for all panels in A, B and C.

**Figure 10:**
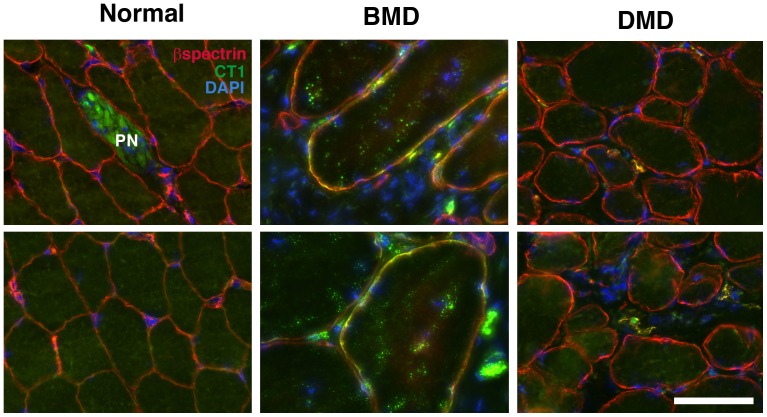
Co-staining of CT1 with βspectrin in BMD muscles. Sections from DMD, BMD and normal human muscle biopsies sections were co-stained with CT1 (green), β spectrin(red) and DAPI (blue). Merged sarcolemmal membrane staining for CT1 and β spectrin is orange-yellow. PN represents intramuscular peripheral nerve. Bar is 50 µm for all panels.

Last, we determined CT carbohydrate expression in DMD and BMD muscles, compared to normal human muscle (Figures 9, 10 and S7). We had previously demonstrated that CT2 shows a binding preference for the CT carbohydrate when Neu5Gc is the sialic acid present, while CT1 shows a binding preference for CT carbohydrates containing Neu5Ac[47]. We first compared CT1 and CT2 staining in DMD (Figure 9A) and normal human (Figure 9B) muscle to determine if CT1 would stain more strongly, as humans do not make Neu5Gc[32] (Figure 9). We also stained mdx muscles (Figure 9C) that had been infected with AAV(rh.74).MCK.*GALGT2* [24] to overexpress the CT carbohydrate. As expected, CT1 stained human muscle, but CT2 did not, while both antibodies stained AAV-*GALGT2*-infected mouse muscle (Figure 9). Normal human and DMD muscles did not show CT1 staining along the sarcolemmal membranes of myofibers, which were co-labeled with β spectrin, but some staining was evident in mononuclear cells (co-stained with DAPI) and also in peripheral nerve (Figure 10). All three BMD muscle biopsies, by contrast, showed increased CT1 staining along myofiber membranes (Figure 10, Figure S7). While it is impossible to generalize with only three cases, we observed at least some CT1-stained positive fibers in all three BMD cases used, and these cases ranged from relatively severe (loss of ambulation at 23) to relatively mild (ambulant at 67). In Figure S7, each BMD case is presented, respectively, with severity ranging from severe on the left to mild on the right.

## Discussion

Both N-glycolylneuraminic acid (Neu5Gc), which requires the *Cmah* gene for its synthesis, and the Cytotoxic T cell (CT) carbohydrate, which requires the *Galgt2* gene for its synthesis, can, when altered, affect the severity of muscular dystrophy in mdx mice; Deletion of *Cmah* [28] or *Galgt2* (Xu et al., in preparation) increases severity of muscular dystrophy in the mdx mouse, decreasing lifespan and sometimes also muscle strength, while increasing *Galgt2* can ameliorate muscular dystrophy, increasing resistance of muscles to injury[24], [55]. Here we have analyzed the expression of these two carbohydrates in patients with DMD and BMD and in GR and GRMD dogs. Both humans and dogs with dystrophin deficiency can show a range of disease severity, however, these phenotypes are generally more severe than those exhibited by the mdx mouse. All human cells lack a functional *CMAH* gene and therefore cannot synthesize Neu5Gc[32], [65]. The data presented here demonstrate that Golden Retriever dog muscles also show little to no Neu5Gc expression (Figs. 1–3), despite lacking a human-like inactivating mutation in the canine *CMAH* gene. The low level of Neu5Gc found in skeletal muscle is consistent with a recent study showing little to no canine *CMAH* gene expression in cell lines made from Western dog breeds[66]. These data support the possibility that reduced Neu5Gc levels in GRMD dog muscles may contribute to their worsened disease severity relative to mdx mice. Similarly, we found very low levels of CT carbohydrate expression in both normal human and DMD muscles (Figure 9 and 10). Surprisingly, BMD muscles show increased membrane expression of CT carbohydrate (Figure 10). The fact that CT carbohydrate is not normally expressed in extrasynaptic membrane of normal, non-diseased, muscle gives an indication that CT carbohydrate may have a unique function in the setting of abnormal dystrophin expression. Because Galgt2 and CT carbohydrate expression are elevated in regenerating muscle, it is possible that elevated CT carbohydrate expression in BMD muscles may relate to some aspect of muscle regeneration. As increased CT carbohydrate can inhibit the development of muscular dystrophy in mdx mice[24], [55], the elevated expression of the CT carbohydrate in BMD muscles, relative to DMD, suggests the potential for GALGT2 to contribute to lessened disease severity in BMD. The expression of a partially functional dystrophin protein, however, would be the primary effector of disease severity in BMD[67].

This study is also the first to describe the cellular and subcellular distribution of Neu5Gc in normal and dystrophin-deficient dog and human skeletal muscles (Figure 7). In addition to low to absent Neu5Gc expression on skeletal myofibers, GRMD muscles expressed Neu5Gc only on a minority of intramuscular satellite cells, CD8+ T lymphocytes and macrophages (Figure 4 and 5). Further, almost no such cells were stained for Neu5Gc in DMD muscles, consistent with loss of CMAH function in humans[32]. By contrast, we did identify intracellular accumulations of Neu5Gc in DMD and BMD skeletal myofibers (Figure 6). This presumably reflects the uptake of Neu5Gc by a salvage pathway from the diet[68], [69]. Neu5Gc levels, for example, are particularly high in red meat[70], and Neu5Gc can be incorporated into cells from ingested glycoproteins in humans[70] and in *Cmah*-deficient mice[57], [69]. Here we have shown that intracellular Neu5Gc co-stains with clathrin and Golgi 58K protein, and to a lesser extent with LAMP1 (Figure 7). This suggests a possible mechanism, much of which has been described previously[68], where internalization of Neu5Gc-containing glycoproteins via endosomes leads to their transport to the lysosome via the sialin transporter. There, Neu5Gc present on glycoproteins could be liberated by lysosomal sialidase. Such a mechanism would ultimately allow recycling of Neu5Gc into CMP-Neu5Gc, transport into the Golgi, and reintegration of Neu5Gc into proteins and lipids by sialyltransferases. The uptake and reincorporation of Neu5Gc into muscle cell proteins may affect muscle disease by altering muscle physiology or by increasing Neu5Gc-driven immune reactions. All humans possess serum antibodies that react with Neu5Gc-containing glycans, and these antibodies could increase damage to Neu5Gc-containing muscle cells[33], [71]. Future work will be required to delineate Neu5Gc's roles and the possible mechanisms controlling its functions.

Our findings in dog skeletal muscle suggest that reduced or absent Neu5Gc in dog muscles may be analogous to *Cmah*-deficient mdx mice, which, like GRMD dogs, have relatively severe muscle disease[28]. GRMD dogs should have no autoimmune response to Neu5Gc, as *Cmah^−/−^*mdx mice [28]and humans can[33], but they may share the loss of function effects of lack of Neu5Gc expression found in *Cmah^−/−^*mdx mice and in DMD patients. The percentage of sialic acid comprising Neu5Gc was reduced in both GR and GRMD muscles by at least 95% relative to mouse muscles (Figure 2). Only 1–2% of total sialic acid was Neu5Gc in any dog muscle studied. Further, Neu5Gc levels in GRMD muscles were reduced relative to GR. This was evident in Neu5Gc measurements of total muscle sialic acid (Figure 2), Neu5Gc immunoblots of muscle glycoproteins (Figure S2) and Neu5Gc immunostaining of muscle sections (Figure 1).

We also wished to determine if Neu5Gc expression plays a role in disease severity of individual dogs and the associated dramatic phenotypic variation. A range of phenotypic features have been used to allow a general definition of disease severity in GRMD dogs[62]. In particular, they have lower tibiotarsal joint (TTJ) tetanic force extension and a plantigrade posture exemplified by more acute tibiotarsal joint angles[16], [19]. Paradoxically, some muscles (especially flexors) undergo early necrosis and then may recover or even hypertrophy. Accordingly, TTJ flexor tetanic force is typically increased in dogs with more severe postural abnormalities[62]. In keeping with this paradoxical functional muscle hypertrophy, the cranial sartorius muscle may become larger as disease progresses (represented by circumference [mm] divided by body weight [kg])[17]. While TTJ flexion does not involve the cranial sartorius muscle, force values tend to track with those of cranial sartorius hypertrophy. Interestingly, muscle membranes of hypertrophied muscle fibers appear to be partially protected against eccentric contraction-induced injury. As a result, the degree of decrement may actually be reduced in dogs with an otherwise severe phenotype (Kornegay JN, unpublished data). If Neu5Gc were playing a role in protecting dystrophic muscles, increased levels might therefore be expected in the VL of mildly affected dogs, which might prevent their subsequent atrophy, or the CS of dogs with a more severe phenotype, which might contribute to increased hypertrophy. With a small number of GRMD dogs in this study, we were unable to draw definitive conclusions regarding the role of Neu5Gc expression in the individual phenotypes.

Similar to our Neu5Gc studies, we have shown that most DMD myofibers have very low to absent CT carbohydrate expression (Figure 9 and 10). By contrast, CT carbohydrate expression was increased in BMD muscles (Figure 10 and S7). Although these observations do not establish a causal relationship, they suggest an association between increased CT carbohydrate and BMD. CT carbohydrate was also increased in some myofibers and macrophages in GRMD muscles (Figure 8), where it may play additional roles. Further work will be required to understand if there is a direct relationship between CT carbohydrate expression and human or canine disease. However, the low expression of Neu5Gc and CT glycans in DMD and GRMD muscles suggests that they do not normally have the potential to ameliorate disease severity in dogs and humans lacking dystrophin protein.

## Supporting Information

Table S1 **GRMD Dog Functional Data (6 Mos).** GRMD dogs have a range of phenotypic features that allow general definition of disease severity.^1^ In particular, they have lower tibiotarsal joint (TTJ) tetanic extension force^2^ and a plantigrade posture exemplified by more acute tibiotarsal joint angles.^3^ Paradoxically, some muscles (especially flexors) undergo early necrosis and then may recover or even hypertrophy. Accordingly, TTJ flexor tetanic force may be increased in dogs with more severe postural abnormalities.^1^ In keeping with this paradoxical functional muscle hypertrophy, the cranial sartorius muscle may be larger (represented by circumference [mm] divided by body weight [kg]).^4,5^ While TTJ flexion does not involve the cranial sartorius muscle, force values tend to track with those of cranial sartorius hypertrophy. Interestingly, muscle membranes of hypertrophied muscle fibers appear to be partially protected against eccentric contraction injury. As a result, the degree of decrement may actually be reduced in dogs with an otherwise severe phenotype (Kornegay JN, unpublished data). For sake of defining disease severity in these GRMD dogs, we characterized several of these features. Ringo and Napoleon were characterized as “moderate/severe”, while Tico, Summer, and Jane were “mild/moderate,” based on TTJ extension tetanic force and TTJ angles below or above (1 N/kg) and (145°), respectively. 1. Kornegay JN, Bogan JR, Bogan DJ, Childers MK, Li J, Nghiem P, Detwiler DA, Larsen CA, Grange RW, Bhavaraju-Sanka RK, Tou S, Keene BP, Howard JF, Jr, Wang J, Fan Z, Schatzberg SJ, Styner MA, Flanigan KM, Xiao X, Hoffman EP: Canine models of Duchenne muscular dystrophy and their use in therapeutic strategies. Mamm Genome 23:85-108, 2012. 2. Kornegay JN, Bogan DJ, Bogan JR, Childers MK, Cundiff DD, Petroski GF, Schueler RO: Contraction torque generated by tarsal joint flexion and extension in dogs with golden retriever muscular dystrophy. J Neurol Sci 166:115–121, 1999. 3. Kornegay JN, Sharp NJH, Schueler RO, Betts CW: Tarsal joint contracture in dogs with golden retriever muscular dystrophy. Lab Anim Sci 44:331–333, 1994. 4. Kornegay JN, Cundiff DD, Bogan DJ, Bogan JR, Okamura CS: The cranial sartorius muscle undergoes true hypertrophy in dogs with golden retriever muscular dystrophy. Neuromuscul Disord 13:493–500, 2003. 5. Kornegay JN, Bogan JR, Bogan DJ, Childers MK, Grange RW: Golden retriever muscular dystrophy (GRMD): Developing and maintaining a colony and physiological functional measurements. In: Duan D (ed). Muscle Gene Therapy: Methods and Protocols. Methods in Molecular Biology, vol. 709, Humana Press, New York, 2011, 105–123.
(DOCX)

Figure S1 **Muscle damage in mild and severely affected GRMD muscles at 6 months of age.** Hematoxylin and eosin staining of muscle cross-sections from GR and GRMD muscles. Cranial sartorius (A–C) and vastus lateralis (D–F) muscles from one normal dog (Heisenberg, A,D), one mildly affected GRMD dog (Summer, B, E) and one severely affected GRMD dog (Napoleon, C, F) are shown. Overall, histopathologic lesions in these GRMD dogs were in keeping with those we and others have described. There was small group muscle necrosis and regeneration in both the cranial sartorius and vastus lateralis. Each muscle had features of necrosis, including hyaline fibers and myophagocytosis (examples are circled). Myofiber mineralization was only seen in the cranial sartorius. Evidence of regeneration, with small basophilic myofibers and numerous central nuclei, was more pronounced in the vastus lateralis. The regenerative response was more mature in the cranial sartorius, with an increase in larger myofibers. Bar is 150 µm for all panels.
(TIF)

Figure S2 **Neu5Gc expression on GR and GRMD glycoproteins relative to mouse.** Western blots of NP-40 extracted muscle protein with anti-Neu5Gc antibody and anti-GAPDH control. Mouse *Cmah^+/+^* and *Cmah^−/−^* skeletal muscle lysates were compared to cranial sartorius (CS) and vastus lateralis (VL) muscles from GR and GRMD dogs that were either mildly or severely affected. Half the amount of mouse protein as dog protein is loaded per lane.
(TIF)

Figure S3 **High expression of sialic acid in GR and GRMD muscle.** *Maackia amurensis* agglutinin (MAA), a lectin that stains α2,3-linked sialic acid, both Neu5Ac and Neu5Gc, was used to stain skeletal muscles from 6 month-old Golden Retriever cross (GR) and Golden Retriever Muscular Dystrophy (GRMD) dogs. Time-matched images are shown. Bar is 200 µm for all panels.
(TIF)

Figure S4 **High sialic acid expression in GRMD muscles at different ages.** Three different GRMD cases (GRMD1, 2, and 3-Napoleon, Jane and Summer respectively) were biopsied and muscles stained for sialic acid using *Maackia amurensis* agglutinin (MAA) at 3, 6 and 13 months of age. Time-matched images of staining of the cranial sartorius and vastus lateralis muscles are shown. Bar is 50 µm for all panels.
(TIF)

Figure S5 **Neu5Gc expression in DMD, BMD and normal human muscle.** Muscle biopsies from Duchenne muscular dystrophy (DMD), Becker Muscular Dystrophy (BMD), or otherwise normal human muscle were immunostained with an antibody specific to *N*-glycolylneuraminic acid (Neu5Gc) or non-immune control sera. Each panel represents a different patient biopsy. Bar is 100 µm for all panels.
(TIF)

Figure S6 **Neuromuscular junction staining of βGalNAc and the CT carbohydrate in GR muscle and elevated CT staining in GRMD muscle.** GR muscle (6mo cranial sartorius) was co-stained with rhodamine-α-bungarotoxin, to label acetylcholine receptors (AChR) concentrated at the neuromuscular junction, and *Wisteria floribunda* agglutinin (WFA), which stains β-linked GalNAc, or CT2, which stains the CT carbohydrate. Below, GRMD muscle (cranial sartorius) was stained with WFA or CT2, compared to control secondary antibody alone. Bar is 100 µm for all panels.
(TIF)

Figure S7 **CT1 immunostaining of DMD, BMD and normal human muscle.** 3 DMD, 3 BMD, and 2 normal human biopsies were immunostained with CT1 and compared to secondary antibody control alone. BMD cases range in severity from severe (loss of ambulation at 23) to milder (ambulant at 36 and ambulant at 67), respectively, from left to right. Bar is 200 µm for all panels.
(TIF)
