# Supplementary material for: A Comparative Study of N-glycolylneuraminic Acid (Neu5Gc) and Cytotoxic T Cell (CT) Carbohydrate Expression in Normal and Dystrophin-Deficient Dog and Human Skeletal Muscle
Source: PLoS One. 2014 Feb 5;9(2):e88226. doi: 10.1371/journal.pone.0088226 (PMC3914967; doi:10.1371/journal.pone.0088226)
Supplement: Table S1 — GRMD Dog Functional Data (6 Mos). GRMD dogs have a range of phenotypic features that allow general definition of disease severity.1 In particular, they have lower tibiotarsal joint (TTJ) tetanic extension force2 and a plantigrade posture exemplified by more acute tibiotarsal joint angles.3 Paradoxically, some muscles (especially flexors) undergo early necrosis and then may recover or even hypertrophy. Accordingly, TTJ flexor tetanic force may be increased in dogs with more severe postural abnormalities.1 In keeping with this paradoxical functional muscle hypertrophy, the cranial sartorius muscle may be larger (represented by circumference [mm] divided by body weight [kg]).4,5 While TTJ flexion does not involve the cranial sartorius muscle, force values tend to track with those of cranial sartorius hypertrophy. Interestingly, muscle membranes of hypertrophied muscle fibers appear to be partially protected against eccentric contraction injury. As a result, the degree of decrement may actually be reduced in dogs with an otherwise severe phenotype (Kornegay JN, unpublished data). For sake of defining disease severity in these GRMD dogs, we characterized several of these features. Ringo and Napoleon were characterized as “moderate/severe”, while Tico, Summer, and Jane were “mild/moderate,” based on TTJ extension tetanic force and TTJ angles below or above (1 N/kg) and (145°), respectively. 1. Kornegay JN, Bogan JR, Bogan DJ, Childers MK, Li J, Nghiem P, Detwiler DA, Larsen CA, Grange RW, Bhavaraju-Sanka RK, Tou S, Keene BP, Howard JF, Jr, Wang J, Fan Z, Schatzberg SJ, Styner MA, Flanigan KM, Xiao X, Hoffman EP: Canine models of Duchenne muscular dystrophy and their use in therapeutic strategies. Mamm Genome 23:85-108, 2012. 2. Kornegay JN, Bogan DJ, Bogan JR, Childers MK, Cundiff DD, Petroski GF, Schueler RO: Contraction torque generated by tarsal joint flexion and extension in dogs with golden retriever muscular dystrophy. J Neurol Sci 166:115–121, 1999. 3. Kornegay JN, [file pone.0088226.s001.docx]

| **Table S1: GRMD Dog Functional Data (6 Mos)** | | | | | | | |
| --- | --- | --- | --- | --- | --- | --- | --- |
| Dog | Genotype | Wt Gain (3-6 Mos) | TTJ  Angle | TTJ Tetanic Flexion (N/Kg) | TTJ Tetanic Extension (N/Kg) | CS Circumference (mm/Kg) | Eccentric  Contraction  Decrement  (1-30) |
| Ringo^*^ | GRMD | 61.4 | 138 | 0.584 | 1.00 | 3.49 | NE |
| Tico^*^ | GRMD | 67.0 | 159 | 0.296 | 3.14 | 2.30 | NE |
| Napoleon | GRMD | 101.6 | 133 | 0.945 | 0.186 | 4.53 | 50.66 |
| Jane | GRMD | 167.7 | 162 | 0.681 | 3.33 | 3.28 | 72.35 |
| Summer | GRMD | 84.9 | 158 | 0.712 | 2.77 | 5.61 | 51.11 |
| Swiper^*^ | Normal | 96.7 | 161 | 1.073 | 4.46 | 1.66 | NE |
| Pedro | Normal | 137.3 | 159 | 1.049 | 3.32 | 1.82 | 11.56 |
| Heisenberg | Normal | 116.7 | 163 | 1.387 | 2.58 | 1.47 | 17.10 |

^*^The method for TTJ torque measurement in Swiper, Tico, Ringo relied on an earlier instrument^2^ and is not directly comparable to the values from the other dogs.
