# Supplementary figures and images for: A Comparative Study of N-glycolylneuraminic Acid (Neu5Gc) and Cytotoxic T Cell (CT) Carbohydrate Expression in Normal and Dystrophin-Deficient Dog and Human Skeletal Muscle
Source: PLoS One. 2014 Feb 5;9(2):e88226. doi: 10.1371/journal.pone.0088226 (PMC3914967; doi:10.1371/journal.pone.0088226)

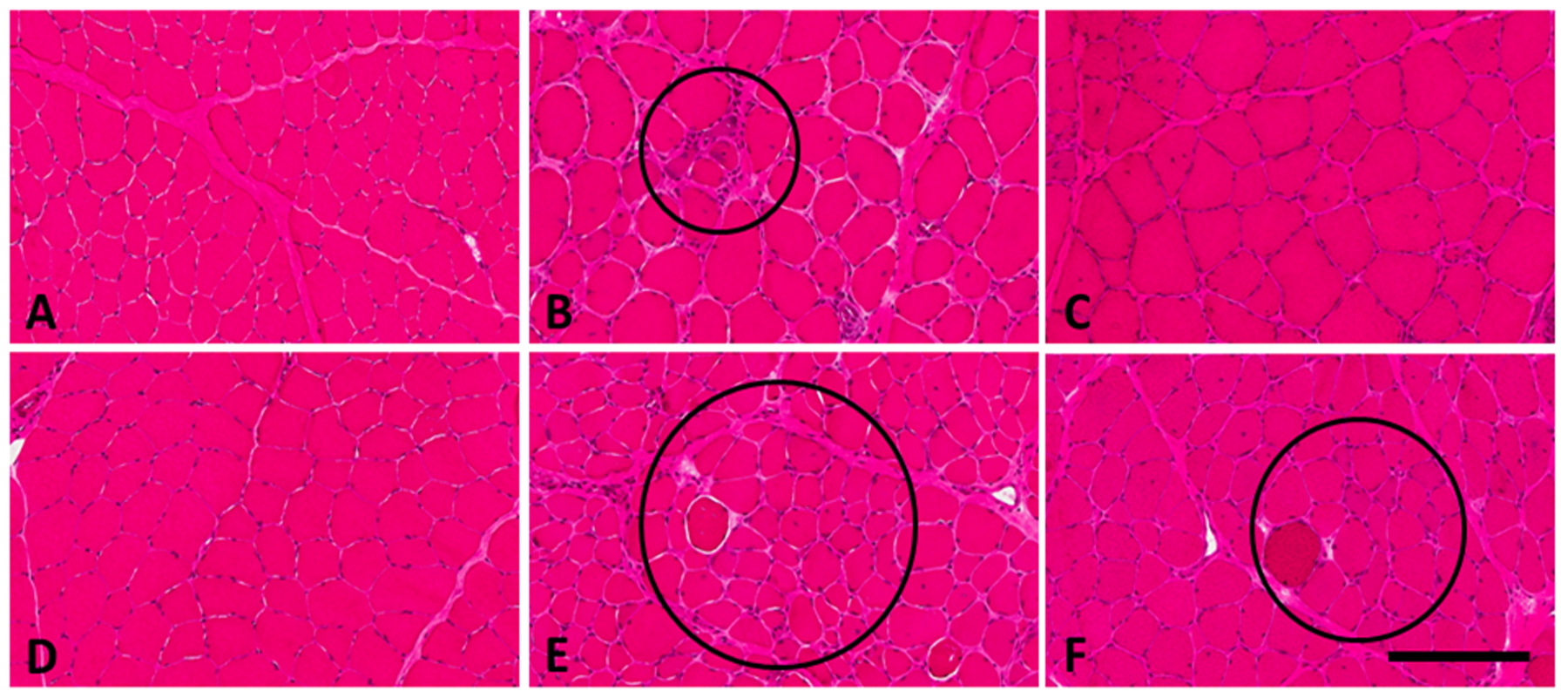

Supplement: Figure S1 — Muscle damage in mild and severely affected GRMD muscles at 6 months of age. Hematoxylin and eosin staining of muscle cross-sections from GR and GRMD muscles. Cranial sartorius (A–C) and vastus lateralis (D–F) muscles from one normal dog (Heisenberg, A,D), one mildly affected GRMD dog (Summer, B, E) and one severely affected GRMD dog (Napoleon, C, F) are shown. Overall, histopathologic lesions in these GRMD dogs were in keeping with those we and others have described. There was small group muscle necrosis and regeneration in both the cranial sartorius and vastus lateralis. Each muscle had features of necrosis, including hyaline fibers and myophagocytosis (examples are circled). Myofiber mineralization was only seen in the cranial sartorius. Evidence of regeneration, with small basophilic myofibers and numerous central nuclei, was more pronounced in the vastus lateralis. The regenerative response was more mature in the cranial sartorius, with an increase in larger myofibers. Bar is 150 µm for all panels. (TIF) [file pone.0088226.s002.tif]

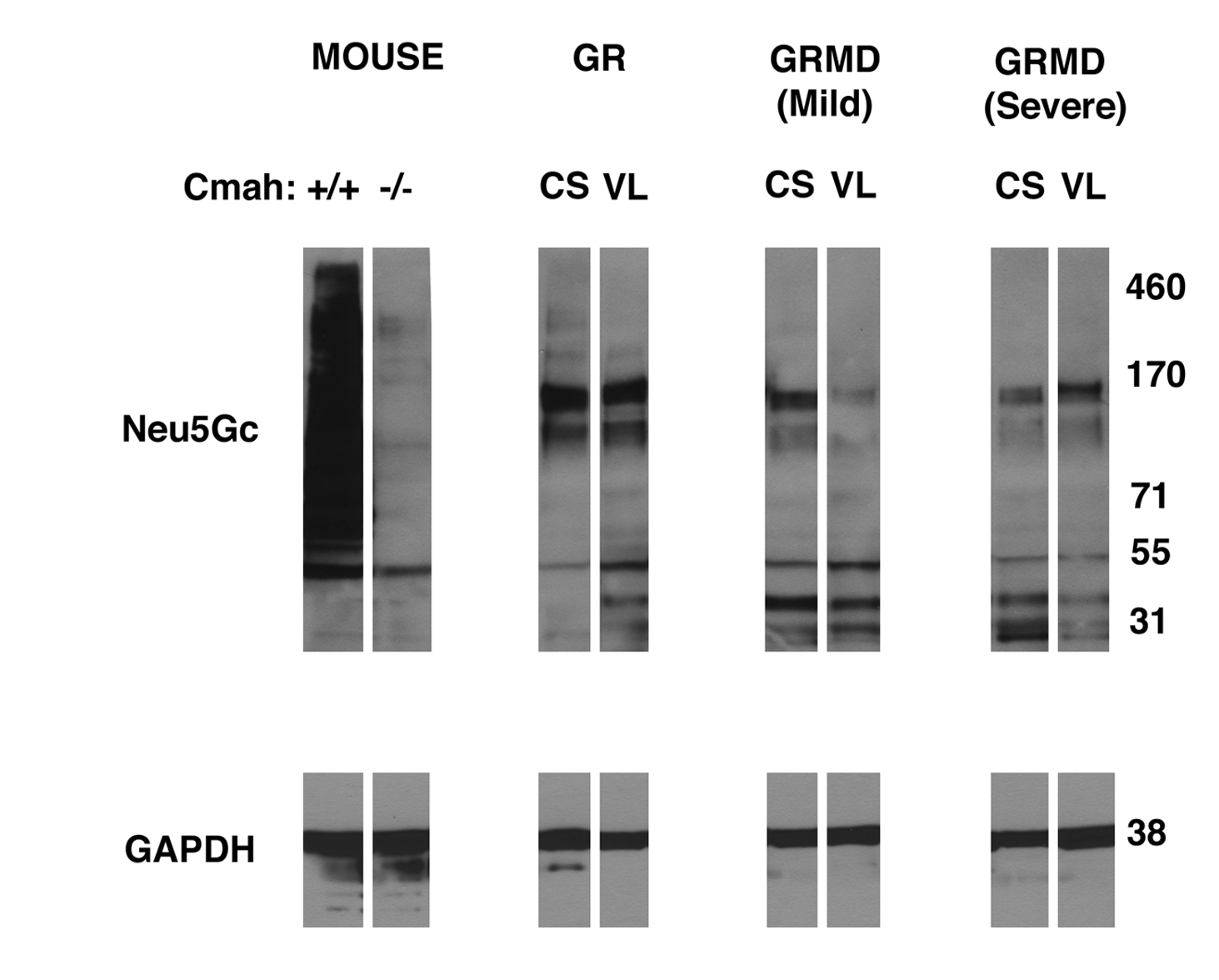

Supplement: Figure S2 — Neu5Gc expression on GR and GRMD glycoproteins relative to mouse. Western blots of NP-40 extracted muscle protein with anti-Neu5Gc antibody and anti-GAPDH control. Mouse Cmah+/+ and Cmah−/− skeletal muscle lysates were compared to cranial sartorius (CS) and vastus lateralis (VL) muscles from GR and GRMD dogs that were either mildly or severely affected. Half the amount of mouse protein as dog protein is loaded per lane. (TIF) [file pone.0088226.s003.tif]

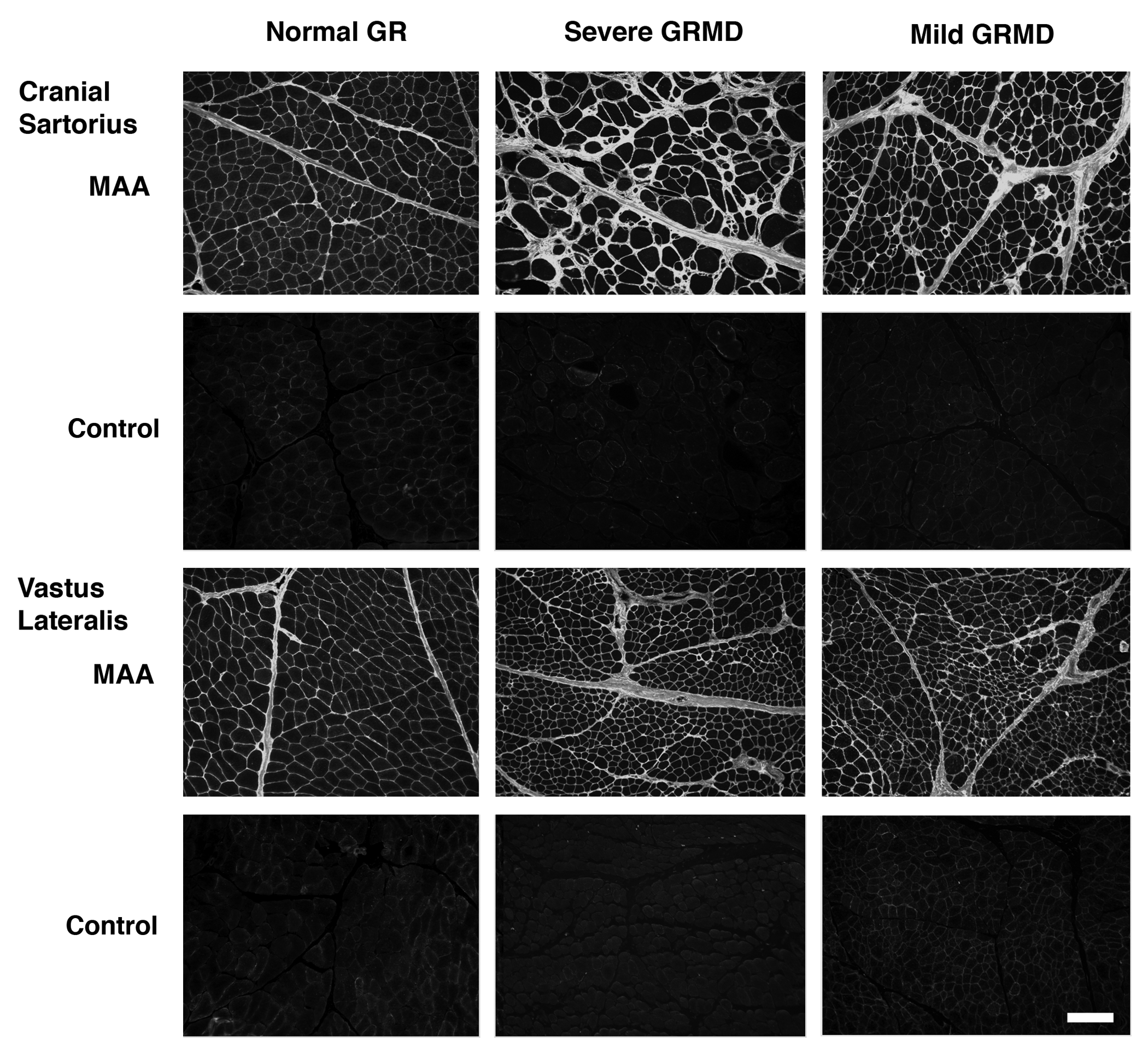

Supplement: Figure S3 — High expression of sialic acid in GR and GRMD muscle. Maackia amurensis agglutinin (MAA), a lectin that stains α2,3-linked sialic acid, both Neu5Ac and Neu5Gc, was used to stain skeletal muscles from 6 month-old Golden Retriever cross (GR) and Golden Retriever Muscular Dystrophy (GRMD) dogs. Time-matched images are shown. Bar is 200 µm for all panels. (TIF) [file pone.0088226.s004.tif]

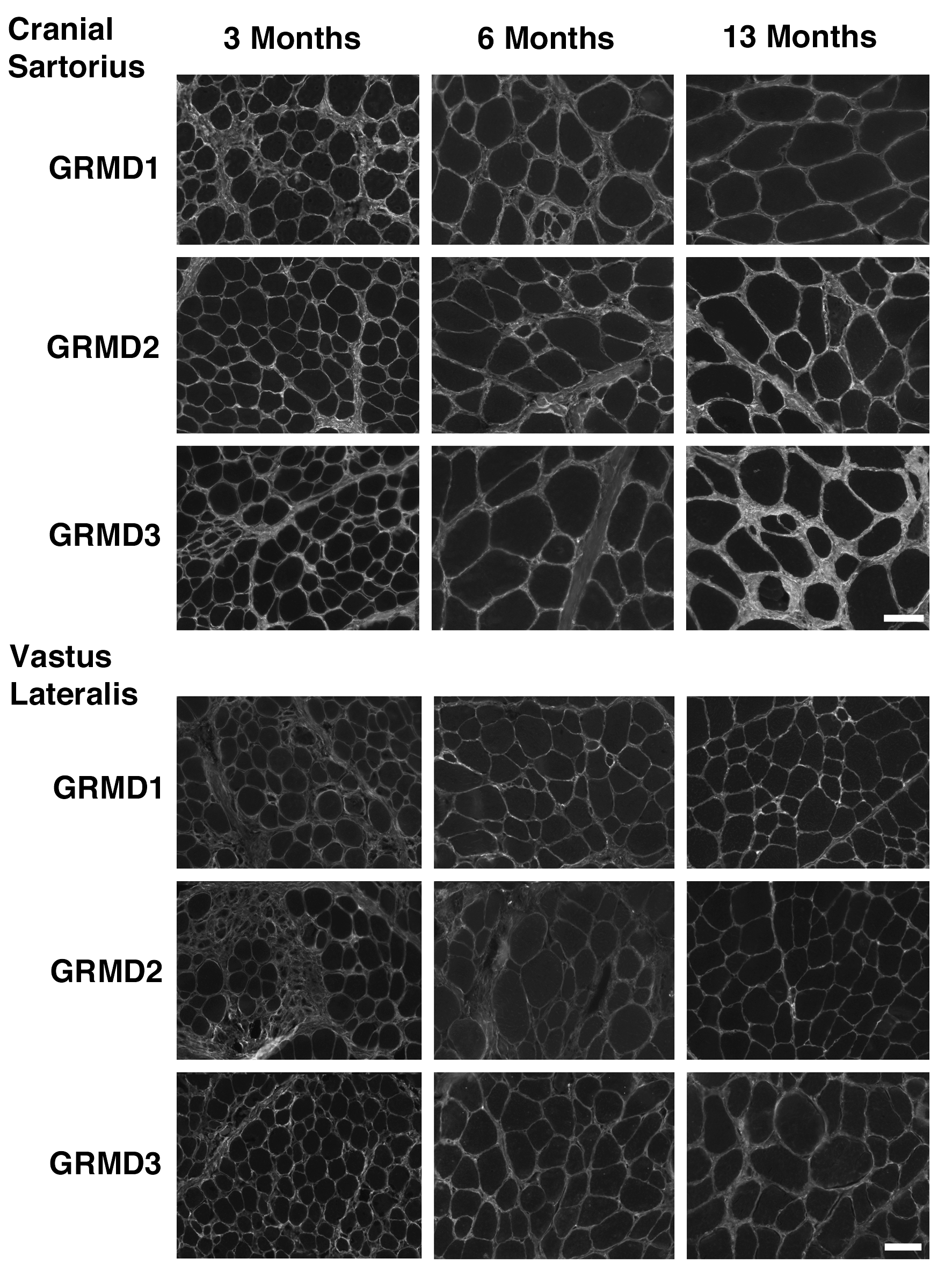

Supplement: Figure S4 — High sialic acid expression in GRMD muscles at different ages. Three different GRMD cases (GRMD1, 2, and 3-Napoleon, Jane and Summer respectively) were biopsied and muscles stained for sialic acid using Maackia amurensis agglutinin (MAA) at 3, 6 and 13 months of age. Time-matched images of staining of the cranial sartorius and vastus lateralis muscles are shown. Bar is 50 µm for all panels. (TIF) [file pone.0088226.s005.tif]

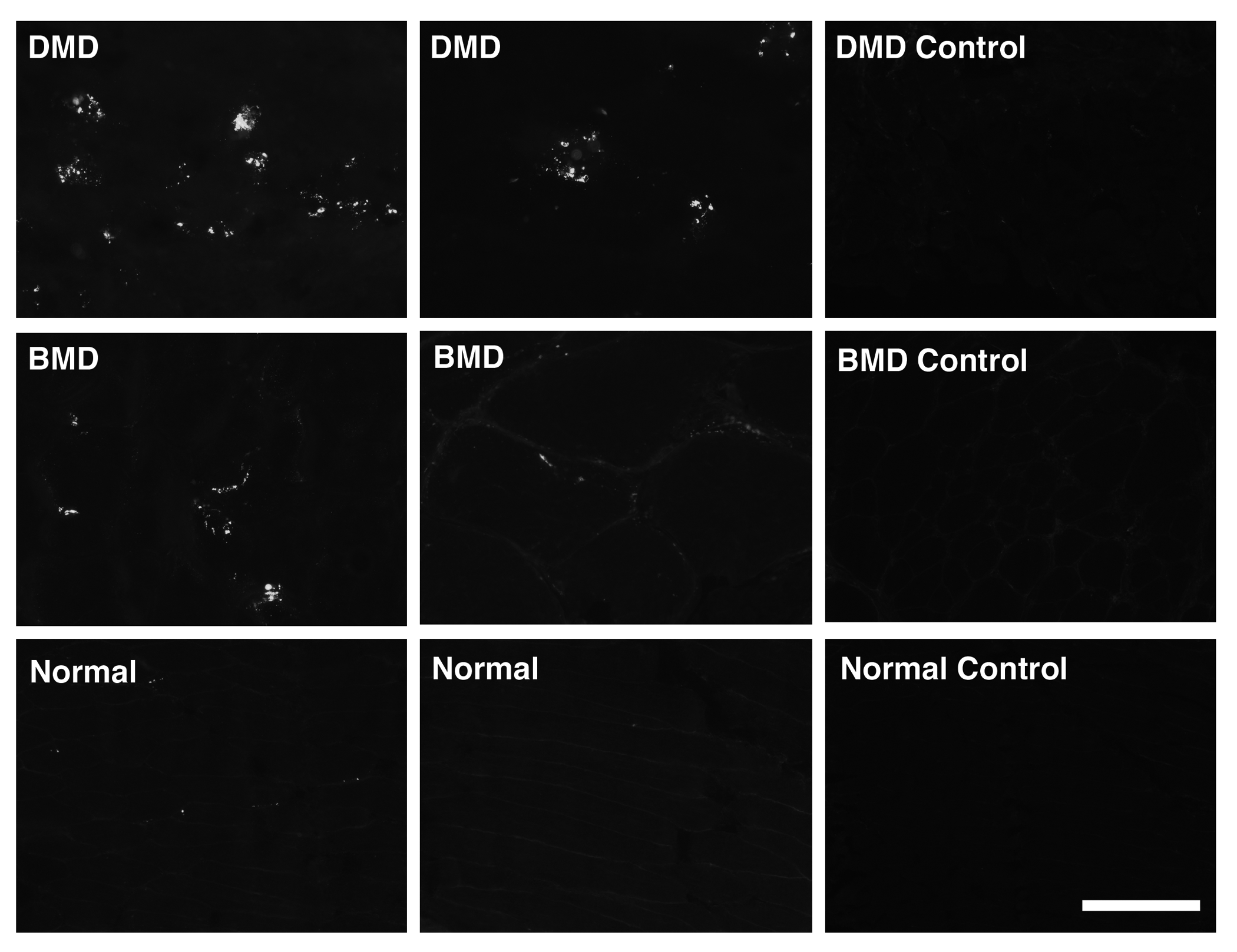

Supplement: Figure S5 — Neu5Gc expression in DMD, BMD and normal human muscle. Muscle biopsies from Duchenne muscular dystrophy (DMD), Becker Muscular Dystrophy (BMD), or otherwise normal human muscle were immunostained with an antibody specific to N-glycolylneuraminic acid (Neu5Gc) or non-immune control sera. Each panel represents a different patient biopsy. Bar is 100 µm for all panels. (TIF) [file pone.0088226.s006.tif]

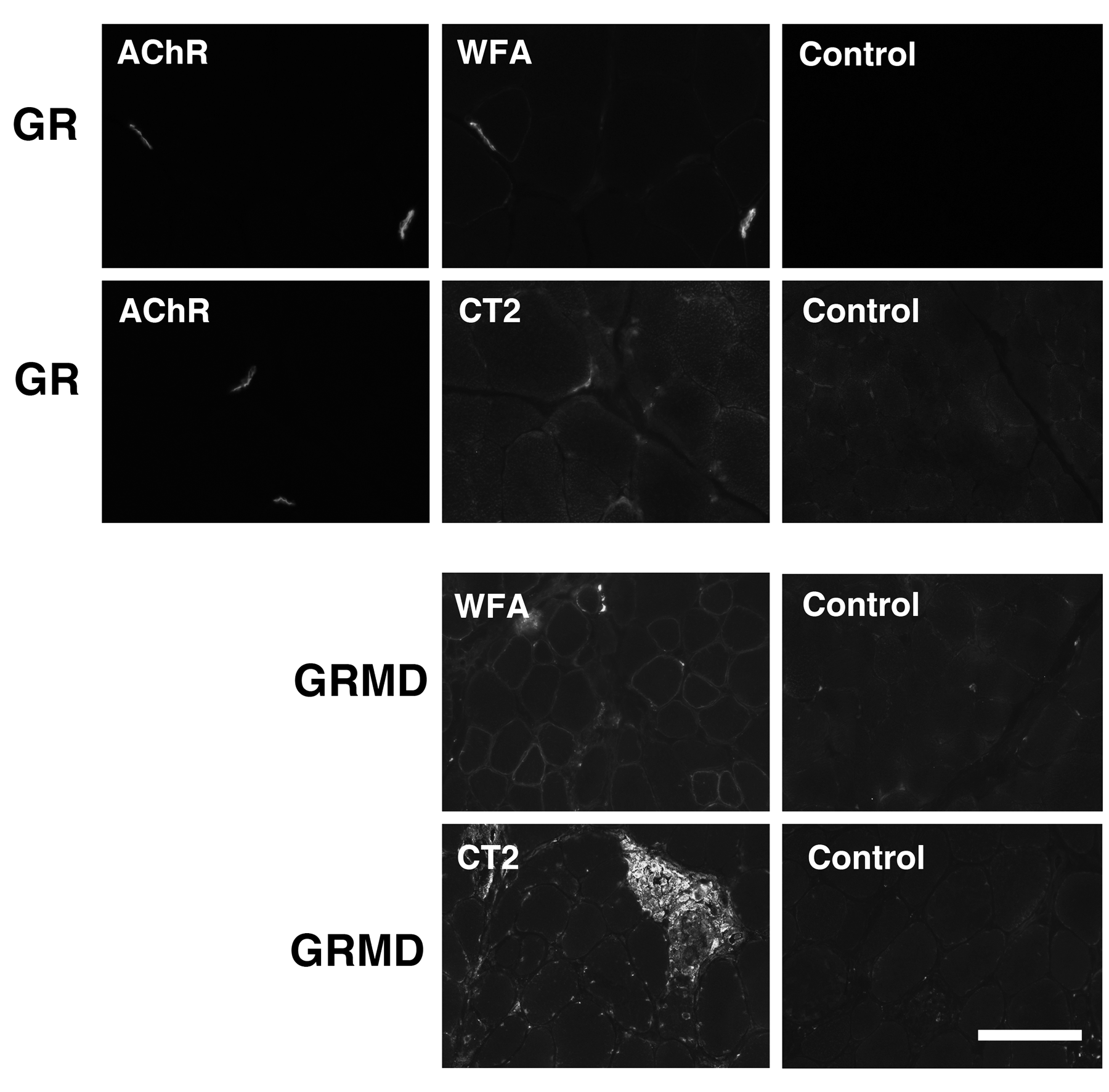

Supplement: Figure S6 — Neuromuscular junction staining of βGalNAc and the CT carbohydrate in GR muscle and elevated CT staining in GRMD muscle. GR muscle (6mo cranial sartorius) was co-stained with rhodamine-α-bungarotoxin, to label acetylcholine receptors (AChR) concentrated at the neuromuscular junction, and Wisteria floribunda agglutinin (WFA), which stains β-linked GalNAc, or CT2, which stains the CT carbohydrate. Below, GRMD muscle (cranial sartorius) was stained with WFA or CT2, compared to control secondary antibody alone. Bar is 100 µm for all panels. (TIF) [file pone.0088226.s007.tif]

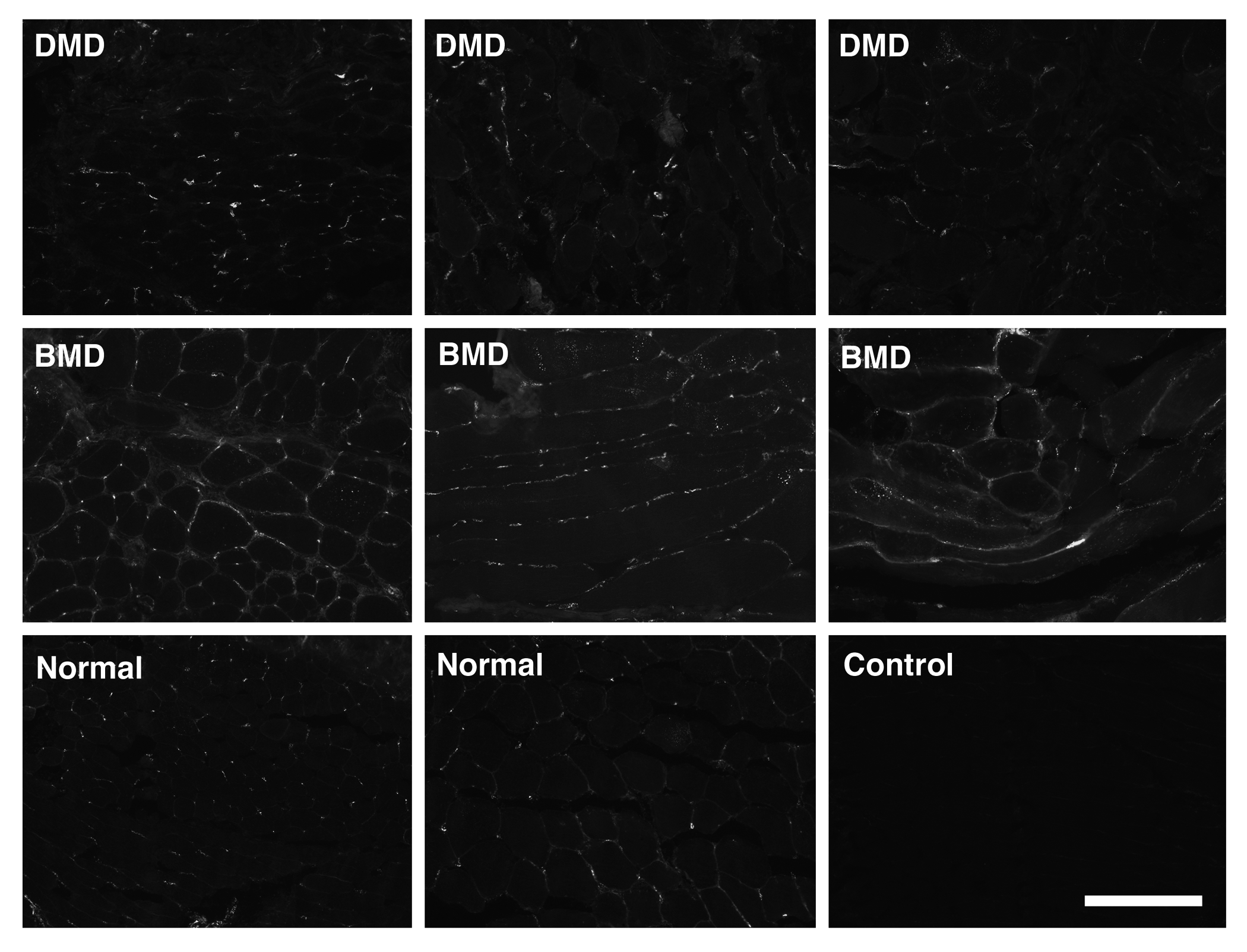

Supplement: Figure S7 — CT1 immunostaining of DMD, BMD and normal human muscle. 3 DMD, 3 BMD, and 2 normal human biopsies were immunostained with CT1 and compared to secondary antibody control alone. BMD cases range in severity from severe (loss of ambulation at 23) to milder (ambulant at 36 and ambulant at 67), respectively, from left to right. Bar is 200 µm for all panels. (TIF) [file pone.0088226.s008.tif]
